# *Tillandsia landbeckii* phyllosphere and laimosphere as refugia for bacterial life in a hyperarid desert environment

**DOI:** 10.1186/s40168-023-01684-x

**Published:** 2023-11-08

**Authors:** Anna Hakobyan, Stefanie Velte, Wiebke Sickel, Dietmar Quandt, Alexandra Stoll, Claudia Knief

**Affiliations:** 1 Molecular Biology of the Rhizosphere, Institute for Crop Science and Resource Conservation (INRES), University of Bonn, 53115 Bonn, Germany; 2 Institute of Biodiversity, Johann Heinrich Von Thünen Institute, Brunswick, Germany; 3 Nees Institute for Biodiversity of Plants, University of Bonn, Bonn, Germany; 4 Centro de Estudios Avanzados en Zonas Áridas Ceaza, La Serena, Chile; 5 Instituto de Investigación Multidisciplinar en Ciencia y Tecnología, Universidad de La Serena, La Serena, Chile

**Keywords:** Phyllosphere, Laimosphere, Bromeliaceae, Hyperarid, Microbiota, Biogeography

## Abstract

**Background:**

The lack of water is a major constraint for microbial life in hyperarid deserts. Consequently, the abundance and diversity of microorganisms in common habitats such as soil are strongly reduced, and colonization occurs primarily by specifically adapted microorganisms that thrive in particular refugia to escape the harsh conditions that prevail in these deserts. We suggest that plants provide another refugium for microbial life in hyperarid deserts. We studied the bacterial colonization of *Tillandsia landbeckii* (*Bromeliaceae*) plants, which occur in the hyperarid regions of the Atacama Desert in Chile, one of the driest and oldest deserts on Earth.

**Results:**

We detected clear differences between the bacterial communities being plant associated to those of the bare soil surface (PERMANOVA, *R*^2^ = 0.187, *p* = 0.001), indicating that *Tillandsia* plants host a specific bacterial community, not only dust-deposited cells. Moreover, the bacterial communities in the phyllosphere were distinct from those in the laimosphere, i.e., on buried shoots (*R*^2^ = 0.108, *p* = 0.001), indicating further habitat differentiation within plant individuals. The bacterial taxa detected in the phyllosphere are partly well-known phyllosphere colonizers, but in addition, some rather unusual taxa (subgroup2 *Acidobacteriae*, *Acidiphilum*) and insect endosymbionts (*Wolbachia*, “*Candidatus* Uzinura”) were found. The laimosphere hosted phyllosphere-associated as well as soil-derived taxa. The phyllosphere bacterial communities showed biogeographic patterns across the desert (*R*^2^ = 0.331, *p* = 0.001). These patterns were different and even more pronounced in the laimosphere (*R*^2^ = 0.467, *p* = 0.001), indicating that different factors determine community assembly in the two plant compartments. Furthermore, the phyllosphere microbiota underwent temporal changes (*R*^2^ = 0.064, *p* = 0.001).

**Conclusions:**

Our data demonstrate that *T. landbeckii* plants host specific bacterial communities in the phyllosphere as well as in the laimosphere. Therewith, these plants provide compartment-specific refugia for microbial life in hyperarid desert environments. The bacterial communities show biogeographic patterns and temporal variation, as known from other plant microbiomes, demonstrating environmental responsiveness and suggesting that bacteria inhabit these plants as viable microorganisms.

Video Abstract

**Supplementary Information:**

The online version contains supplementary material available at 10.1186/s40168-023-01684-x.

## Introduction

Hyperarid deserts such as the Atacama Desert in Chile are largely devoid of macroscopic life, especially in the inner core, where available water has been reported to be below the limit for photosynthetic activity and therewith for plant growth [1]. The lack of water also limits microbial life in such deserts. Besides strong water limitation, microorganisms have to cope with a combination of high-temperature fluctuations, strong UV radiation, and in part saline soil conditions [2]. Consequently, habitats such as soils, which are consistently colonized by microorganisms and often host the most diverse microbial communities on Earth [3], are strongly depleted of microorganisms in hyperarid deserts [4–6]. Strong changes in bacterial community composition have been observed particularly in the transition from arid to hyperarid conditions, commonly characterized by an enrichment of *Actinobacteria* and in part *Chloroflexi*, whereas common soil bacteria such as *Proteobacteria* become often depleted, especially in surface soils [4, 7, 8]. Besides, altered community assembly processes have been reported with increasing aridity [9]. Thus, specialized microbial communities of limited diversity and substantially reduced in abundance and activity are found in this habitat [6]. Microbial life rather flourishes in specific refugia, which support highly specialized microbial communities. These can be observed beneath translucent quartz stones, underneath rocks, or within halite crusts (e.g., [1, 2, 10–12]). Desert plants might represent another refugium of microbial life hosting specifically adapted microorganisms, but this has so far not been studied for plants growing under hyperarid conditions.

In general, plants are well-known to be colonized by microorganisms, aboveground in the phyllosphere as well as belowground in the rhizosphere [13, 14]. Nonetheless, the bacterial communities of plants have only been analyzed in a few studies in semiarid or arid landscapes, including work on *Acacia* and *Tamarix* trees, *Agave* and *Atriplex* species, or cacti [15–20]. These studies reported differences in the microbial community composition in different plant compartments and plant species, temporal changes, or biogeographic patterns. Plants growing in hyperarid environments such as the Atacama Desert have so far only been studied with focus on the rhizosphere microbiota [21–24], while the phyllosphere microbiota has not been studied yet. Consequently, it is not known whether the phyllosphere provides another refugium for microbial life in this extreme environment and whether a typical phyllosphere microbiota can establish even under these conditions.

**Fig. 1:**
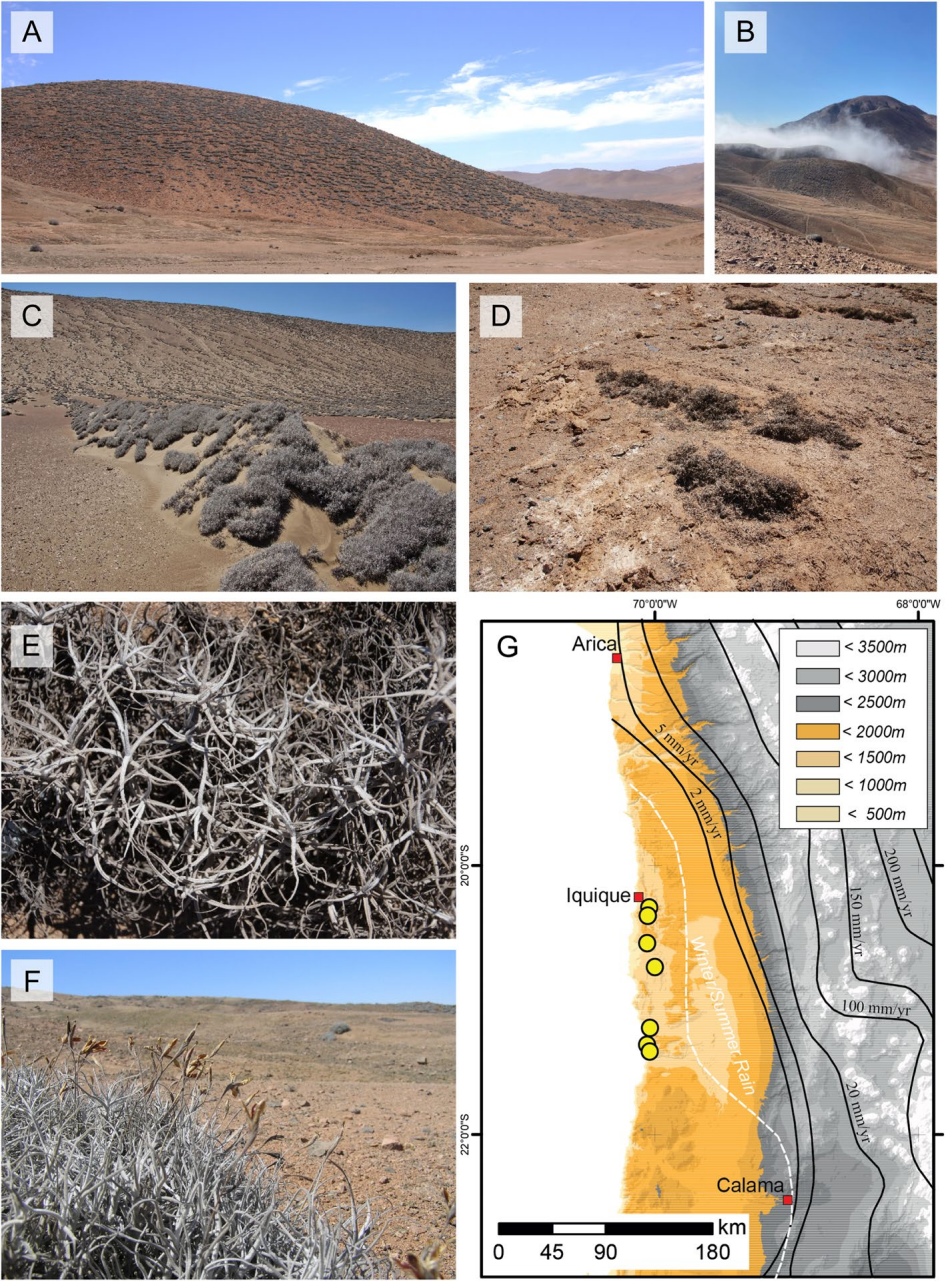
*Tillandsia landbeckii* populations in the Atacama Desert. **A** Plants of the Cerro Chipana population growing on a southwest facing hillslope. **B** Incoming fog at Cerro Chipana. **C** Salitrera San Lorenzo 2 population on a southwest facing hillslope and on sand dunes. **D** Partially dead plants of the Cerro Peninsula population. **E** and **F **Shoots and inflorescences of *T. landbeckii*. **G** Map of the Atacama Desert with elevation being color coded and sampling sites highlighted by yellow circles. Mean annual rainfall is indicated by black lines

The Atacama Desert in Chile is considered as one of the driest deserts on Earth with plants occurring primarily along the eastern and western margins, where they are supported by sporadic rainfall or fog [25–27]. One particular plant genus being well adapted to hyperaridity in this desert is *Tillandsia*, a member of the *Bromeliaceae* family. Populations of the most widely distributed species, *Tillandsia landbeckii*, occur along the Coastal Cordillera, where they form so-called *Tillandsia* lomas (Fig. 1A) [28]. These vegetation formations occur mostly as spatially isolated generally monospecific populations in the Chilean and Peruvian coastal desert systems, although occasionally few other species of the genus might be intermingled at a low density [29, 30]. In Chile, *Tillandsia* lomas occur from 3 to 45 km inland at an elevation of 900–1300 m [28, 31], where rainfall has been reported to be < 2 mm y^−1^ (Fig. 1G), therewith representing the driest part of the desert [32]. *Tillandsia* plants are primarily supported by fog, which reaches the lomas in corridors from the coast (Fig. 1B) [28, 33]. They often form parallel bands, perpendicular to fog penetration (Fig. 1C) [28, 34]. They obtain fog water by water-absorbing trichomes, which cover their leaves, rather than by functional roots, which are usually lacking [33, 35, 36]. Nutrients such as nitrogen are considered to be also largely fog supplied [37, 38], whereas the lower parts of the shoots are usually buried under sandy substrate, therewith supporting anchorage [29]. Such belowground shoots are not considered as rhizosphere but have been termed laimosphere [39]. This compartment has not gained much attention in microbiome research so far but very likely represents a different habitat compared to the phyllosphere or the rhizosphere of other plants. A very first recent study reports that *T. landbeckii* supports microbial life in the soil beneath the plants, as this soil showed increased bacterial abundance and diversity compared to the bare soil [40]. However, the plant itself has not been analyzed in that work, neither above- nor belowground.

The aim of this study was to analyze the microbiota associated with *T. landbeckii* plants, which are adapted to grow under the driest conditions on Earth. Plant populations sampled in this study were located in regions with an aridity index ≤ 0.0125 [41] or rainfall ≤ 1 mm y^−1^ [38]. The overarching question was whether *T. landbeckii* provides specific habitats for bacteria in a hyperarid desert environment and supports microbial life. To evaluate this, we addressed the following more specific questions:

1. Are *T. landbeckii* plants colonized by a specific bacterial community that is distinct from its surroundings?
2. Do we see habitat differentiation, i.e., does the phyllosphere host a different bacterial community compared to the laimosphere?
3. Do biogeographic patterns exist in the *T. landbeckii*-associated bacterial communities?
4. Does the plant-associated bacterial community undergo seasonal (temporal) variation?

To address these questions, we collected shoots of *T. landbeckii* plants from seven different locations in the Atacama Desert in northern Chile. The plant-associated bacterial community was compared to the surface soil community to assess habitat specificity. We compared the bacterial community of the plant phyllosphere to the laimosphere in order to evaluate the capacity of *T. landbeckii* to offer different habitats for bacteria in a hyperarid desert and to host microbial communities resulting from specific selection processes. To assess seasonality, selected sampling sites were visited twice. In all samples, bacterial community composition was analyzed based on 16S rRNA gene-based amplicon sequencing.

## Material and methods

### Study sites and sample collection

Samples were collected from seven different *T. landbeckii* populations [31] in October 2016 (spring) and March 2017 (autumn) in the northern Chilean Atacama Desert (Fig. 1G and Table 1). As phyllosphere samples, shoots were collected from ten individual plants per study site. Collection occurred along a zigzag path within an area with a radius of approx. 15 to 110 m, depending on the population size at the respective site. Five to ten laimosphere samples were collected from the same plant individuals that were selected for taking phyllosphere samples (Table S1). In addition, dead plant material from two to five plants was collected above- or belowground at two sites (Salar Grande, Cerro Pajonal) in 2016. Furthermore, three to four barren soil surface samples (0–1 cm depth) were collected per site. These were taken near the first, middle, and last plant collected at each site and thus coincide with the spatial area of the sampled plant population.

**Table 1:** Location of study sites from south to north and sampling dates

Site	Population ID^a^	Latitude, longitude	Elevation	Sampling dates
Alto Chipana — Rio Loa	4	S21°21′18″ W70°00′31″	936–955	21 October 2016 20 March 2017
Alto Chipana	5	S21°18′08″ W70°01′39″	992–1014	21 October 2016 20 March2017
Salar Grande	6	S21°10′47″ W70°00′30″	902–951	22 October 2016
Cerro Pajonal	7	S20°43′33″ W69°58′13″	967–970	23 October 2016 18 March 2017
Salitrera San Lorenzo 2 (Oyarbide)	8	S20°31′45″ W70°02′08″	1077–1087	18 March 2017
Cerro Guanaco	11	S20°20′07″ W70°01′56″	1043–1055	24 October 2016 17 March 2017
Cerro Carpas	12	S20°17′07″ W70°00′49″	998–1028	24 October 2016

^a^Population ID as given by Merklinger et al. (2020) [31]

For the phyllosphere samples, several plant shoots per plant individual were manually plucked and transferred into paper bags. These were placed on silica in sealable plastic bags to dry the plant material. Silica was replaced during the drying process when needed. For the laimosphere samples, buried plant shoots were pulled from the ground and shaken to remove loosely attached sand particles before bagging. The buried shoots were up to 15 cm long and were buried in sand to a maximum depth of approx. 10 cm. Surface soil samples were collected with a small shovel and transferred into sealable plastic bags. All samples were shipped to Germany for further processing. Considering that all sample material was completely dry for shipping, we do not expect that substantial changes occurred in the microbiota due to shipment at ambient temperature. However, we cannot fully exclude that some changes were introduced during the drying process of vital plant material.

### Sample processing and DNA extraction

Dry plant material was crushed under a laminar flow using sterile mortars and afterwards stored in 15-mL tubes at −20 °C until DNA extraction. For DNA extraction, 0.1 g of the homogenized material was transferred to Lysing Matrix A tubes provided with the FastDNA SPIN Kit for DNA extraction (MP Biomedicals). Extraction was performed following the manufacturer’s instructions with the following modifications. To rehydrate the material, 300 µL of sterile water was added, and the samples were incubated for 2–3 h at 4 °C. The kit-supplied cell lysis solution CLS-VF and protein precipitation solution PPS were added, and bead beating was performed in the FastPrep instrument for 90 s twice for lysis. The matrix-bound DNA was additionally purified by removing the supernatant after brief centrifugation at 14.000 × g and resuspension in 1 mL of 5.5-M guanidine thiocyanate solution. The matrix was again pelleted by centrifugation and then resuspended in 600 µL of guanidine thiocyanate solution to load the matrix onto the kit-supplied spin filter for further processing. DNA elution was performed with two times 50 µL of DES elution solution.

DNA extraction from surface soil was most successful with the NucleoMag DNA Microbiome kit (Macherey–Nagel). Extraction was performed from 0.5 g of soil following the instructions of the kit with the following modifications: (i) the volume of the kit-supplied lysis buffer MI1 was increased to 800 µL; (ii) the lysates were additionally incubated with lysozyme (50 mg/mL) for 30 min at 37 °C, followed by incubation with proteinase K (20 mg/mL) for 30 min at 55 °C; (iii) elution of DNA was done in two steps (2 × 50 µL PCR grade water) resulting in 100 µL total eluate for each sample; and (iv) DNA was concentrated using vacuum drying. As extraction controls in both procedures, sterile tubes with only ceramic beads were used and treated similarly to the plant and soil samples. DNA concentration in all extracts was quantified using the QuantiFluor dsDNA System (Promega Corporation, Fitchburg, WI, USA), and DNA was stored at −20 °C for further use.

### 16S rRNA gene PCR and sequencing

For bacterial community analyses, 16S rRNA gene amplicons were generated in a nested PCR approach similarly as described by Becker et al. [42]. DNA was first amplified using a LNA PCR protocol with modified primer set 63f/1492r to suppress the amplification of plant organelle-derived 16S rRNA genes [43]. The 25-μL PCR reaction mixture consisted of 5 µL of 5 × polymerase buffer, 0.25 µL of each primer [25 mM], 0.25 μL of BSA [0.8 µg/µL], 0.25 µL of dNTP mixture [25 mM of each dNTP], 1 µL of MgCl_2_ [50 mM], 0.25 μL of Herculase II Fusion DNA polymerase (Agilent Technologies), and 3 μL of DNA template and PCR-grade water (Qiagen). The PCR reaction was conducted upon initial denaturation at 95 °C for 4 min with 30 cycles (95 °C, 30 s; 70 °C, 30 s for LNA primer annealing; 56 °C, 30 s for 63f/1492r primer annealing; 72 °C, 45 s), followed by a final elongation at 72 °C for 10 min. A subsequent nested PCR was performed using the primers 799f/1193r (V5–V7 region) with sample-specific 8-mer barcodes in 3 × 50-μL assays to obtain enough PCR product for the downstream cleanup steps. Here, 5 μL of PCR product from the first round was used as template and applying a temperature profile consisting of initial denaturation at 95 °C for 4 min, 15 cycles (95 °C, 30 s; 45 °C, 30 s; 72 °C, 30 s) and final elongation as before. The three technical replicates of each sample were pooled prior to DNA quantification using the QuantiFluor dsDNA system (Promega). PCR products of the different samples were pooled at equimolar ratios, and the resulting pool was purified and concentrated using the HighPrep PCR Clean-up System kit (MagBio Genomics, Gaithersburg, MD, USA). The pooled PCR products of the correct size were purified from agarose gel using the QIAEX II gel extraction kit (Qiagen) to eliminate traces of unspecific products. Library preparation and sequencing on a NovaSeq system (Illumina) were done by Novogene (UK), generating paired-end reads (2 × 250 bp).

### Sequence data analysis

The raw sequence reads were processed as described by Becker et al. [42]. In brief, the data were demultiplexed, primers removed, and reads further processed with QIIME2 version 2022.2 [44]. The classified reads were quality filtered by removing rare amplicon sequence variants (ASVs) that appeared less than 20 times and in less than five of the 156 successfully sequenced samples (Table S1). Likewise, samples with less than 10,000 reads were excluded. Two of the samples were removed as a result of these filtering parameters. Quality-filtered reads (25,922,551 sequences) were processed using default parameters of DADA2 [45] implemented in QIIME2 and grouped into 1457 ASVs, ranging from 17,981 to 1,083,504 sequences per sample (Table S1). The taxonomic assignment of ASVs was done using a custom *classify-sklearn* plug-in classifer against the SILVA SSU138 Ref NR99 database by sub-setting to the amplicon region and using the last common ancestor method [46–48]. ASVs classified as mitochondria, chloroplasts, and Eukaryota were removed (1.3% of assigned ASVs). This dataset consisted of 1438 ASVs and was further analyzed using the *phyloseq* R package (version 1.40.0) [49].

As DNA extraction controls and PCR-negative controls of the surface soil samples were slightly positive according to agarose gel electrophoresis, they were included in sequencing. The ASVs resulting from these controls were removed from the soil samples following the prevalence-based approach of the *decontam* R package (version 1.16.0) [50]. Here, the prevalence (presence/absence across samples) of each ASV in true-positive samples was compared to the prevalence in negative controls to identify the contaminants. Both extraction and negative controls were used to assess the prevalence of the contaminant ASV sequences in the surface soil samples. The threshold was set to 0.5, therewith identifying all sequences as contaminants that were more prevalent in negative/extraction controls than in positive samples. This resulted in the removal of 103 ASVs. Additionally, all reads representing *Aquabacterium*, which dominated in the DNA extraction control but were not fully removed by *decontam*, were manually removed in *phyloseq* (301 ASVs). The remaining 1034 ASVs were used for final data analyses (Table S2). We rate the surface soil samples of sufficient quality for the analysis we present and conclusions we draw, even though the extraction control remained similar to some of the soil samples (Figure S1).

### Statistical data analysis and visualization

Statistical analyses were performed in QIIME2 and R (version 4.2.0) [51]. Analyses were first done on the complete dataset including all samples from phyllosphere, laimosphere, and surface soil. More detailed analyses were then performed with data subsets to compare phyllosphere vs. laimosphere, living vs. dead plants (Fig. 1C and D), and spatial and seasonal dynamics. Alpha diversity was estimated by Shannon’s diversity index using a feature table rarefied to 10,000 reads per sample. Additionally, evenness and Faith’s PD indices were analyzed in selected datasets upon significant results from Shannon’s diversity. A Kruskal–Wallis test was applied to test for significant differences, followed by Dunn’s test with Benjamini–Hochberg correction for multiple testing. For data comparisons between phyllosphere vs. laimosphere and seasonal dynamics in the phyllosphere, a Wilcoxon signed-rank exact test was applied for paired sample sets and a Wilcoxon sum exact test for some non-paired sample sets (e.g., Cerro Pajonal 2016 vs. 2017).

Differences in bacterial community composition were evaluated by principal component analysis using the non-rarefied dataset upon robust-centered log-ratio (rCLR) transformation [52]. This analysis was performed with the R package *vegan* (version 2.6–2) [53]. Statistical differences were calculated by *adonis2* function (*vegan* package) on a robust Aitchison distance matrix, which is a form of one-way permutational multivariate analysis of variance (PERMANOVA). This was followed by pairwise comparisons (*pairwiseAdonis* version 0.4) [54] using Benjamini–Hochberg correction for multiple testing. For the comparison of phyllosphere to laimosphere, pairwise differential abundance analysis at ASV level was performed using analysis of compositions of microbiomes with bias correction (ANCOM-BC) with detection for structural zeros turned on [55]. Conservative variance estimates of the test statistic were used, and *p*-values were adjusted using Holm’s correction (*p*_*adj*_). All ASVs with *p*_*adj*_ ≤ 0.05 and log_2_ fold changes of ≥ 2 or ≤ −2 were considered as significantly differentially abundant.

The visualization of the data was done in R using *ggplot2* (version 3.3.6) [56]. A Venn diagram was generated with the *ps_venn* function of the *MicEco* package (version 0.9.18) [57]. The heat trees were created within the *metacoder* package using the *heat-tree* function [58]. Here, the number of reads in each taxon was calculated, and the samples were grouped by phyllosphere or laimosphere. The abundance value of 0.001 was set as threshold to remove the low-abundance taxa and reduce the complexity of the plots.

## Results and discussion

### Compartment-specific colonization of *T. landbeckii* plants

**Fig. 2:**
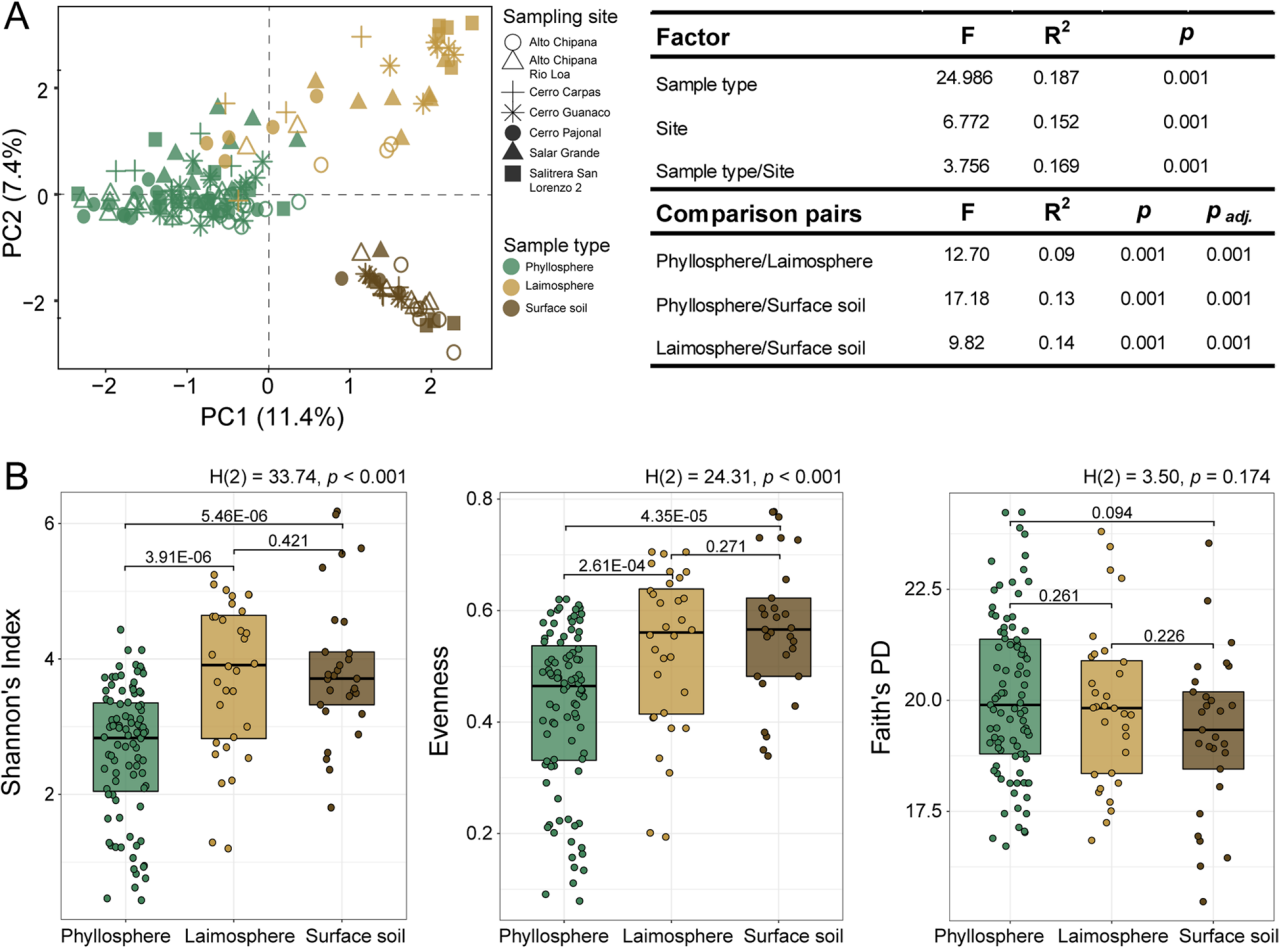
Differences in bacterial community composition between phyllosphere, laimosphere, and bare soil surface samples. **A** Principal component plot (of rCLR transformed data) showing differences between the three sample types (symbol color) and of the seven different *T. landbeckii* populations (symbol shape). Significant differences between groups of samples were evaluated based on PERMANOVA on a robust Aitchison distance matrix and are listed besides the plot. **B** Differences in Shannon’s index, evenness, and Faith’s PD. Overall differences were assessed based on Kruskal–Wallis tests, followed by Dunn’s test with Benjamini–Hochberg correction for pairwise comparisons

PCR amplification and sequencing were successful for the majority of phyllosphere and laimosphere samples (Table S1), indicating that bacteria were associated with these plants. To evaluate whether these two plant compartments host specific bacterial communities, we analyzed the associated bacterial communities comparatively to the community structure in the surface soil. Both the soil surface and the phyllosphere are assumed to receive bacteria from atmospheric deposition processes and should be similar in case deposition would be the major process leading to an accumulation of bacterial cells in these habitats. Differentiation of bacterial communities in dependence on the two plant compartments and surface soil samples was evaluated by principal component analysis (PCA) using the amplicon data of all samples (Fig. 2A). This revealed a clear separation of samples due to the origin from phyllosphere, laimosphere, and the soil surface, especially along the first axis of the plot, which covered 11.4% of the variation. The distinctiveness by compartment was confirmed by PERMANOVA (*R*^2^ = 0.187, *p* = 0.001). Subsequent pairwise PERMANOVA resulted in significant differences between all three sample types with the soil surface being most distinct to phyllosphere (*R*^2^ = 0.13, *p*_*adj*._ = 0.001) and laimosphere (*R*^2^ = 0.14, *p*_*adj*._ = 0.001) (Fig. 2A). Thus, it can be concluded that *T. landbeckii* provides distinct habitats for bacteria in comparison to the surface soil. The leaf-associated microbiota is thus most likely not the result of mere atmospheric deposition, but instead the consequence of selective processes, leading to the establishment of a characteristic phyllosphere microbiome. This is further supported by the finding that the laimosphere was also clearly distinct from the phyllosphere, indicating that this represents another specific habitat for microorganisms.

Differences were also observed in alpha diversity with lower Shannon’s index in the phyllosphere than in the laimosphere and soil surface (*p* < 0.001) (Fig. 2B). While evenness showed the same pattern, Faith’s PD, informing about the phylogenetic diversity by considering phylogenetic distances of the detected taxa, was not significantly different between the three sample types. Thus, the observed differences in Shannon’s index are primarily the result of lower evenness in the phyllosphere, while phylogenetic diversity was similar in all three compartments. This is remarkable, because bacterial diversity in the phyllosphere is usually substantially lower than in soil and also lower than in the rhizosphere [14, 59]. Plants in hyperarid deserts may thus be able to support a relatively diverse microbiota compared to their surroundings, even though it has to be kept in mind that the soil surface may be particularly depleted in diversity [4].

At this point, it should be noted that the use of different DNA extraction procedures for phyllosphere and laimosphere samples compared to soil samples may have contributed to observed differences between these sample types. However, the possible method-related variation can be considered to be much smaller than the clear sample-type-specific differences we observed. The use of different kits was necessary to obtain sufficient DNA of adequate quality from the different sample types.

**Fig. 3:**
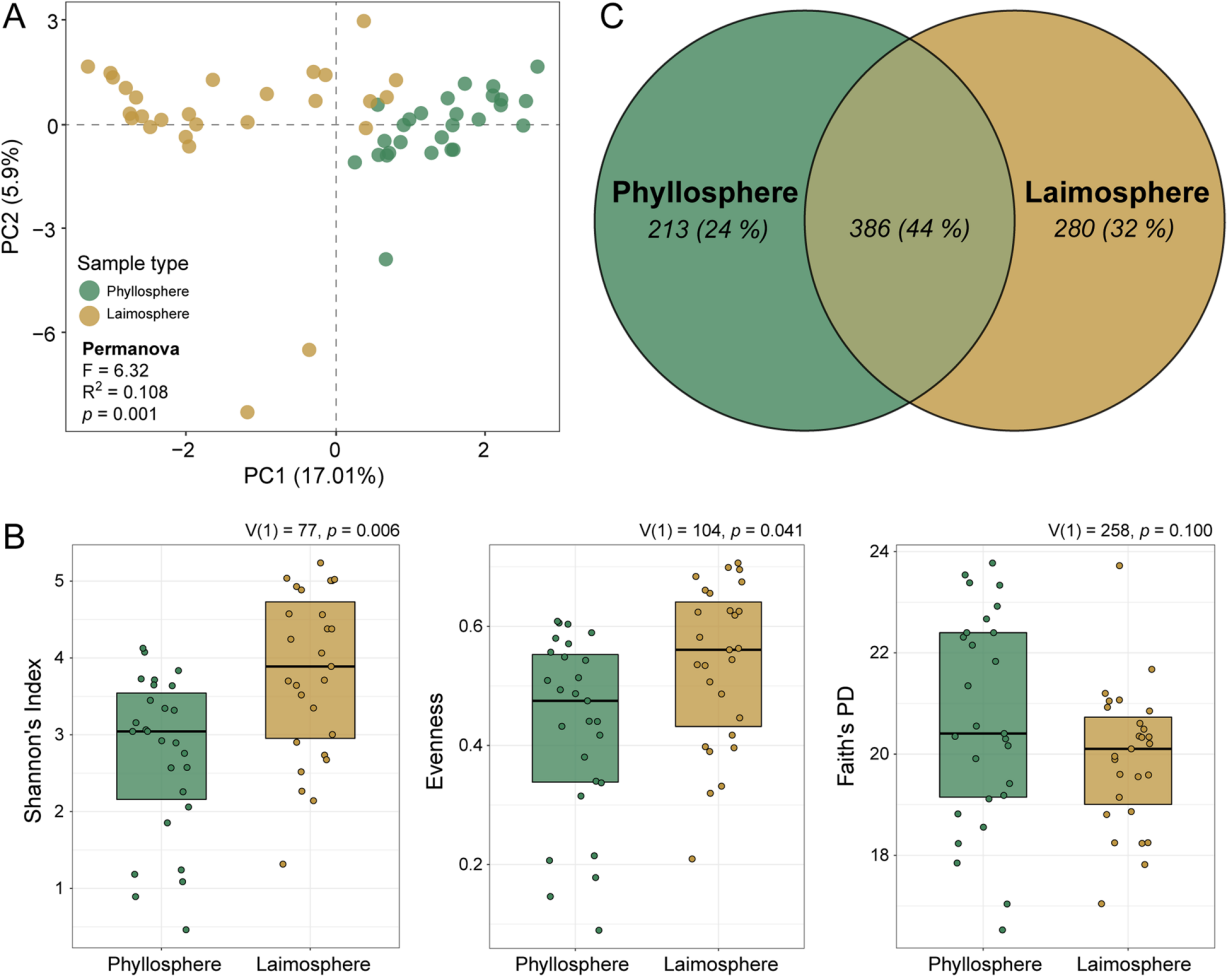
Differences in bacterial community composition between phyllosphere and laimosphere with focus on those 27 plant individuals that were sampled for both compartments. **A** Principal component plot (rCLR transformed) illustrating beta diversity. Differences related to compartment were evaluated by PERMANOVA on a robust Aitchison distance matrix. **B** Differences in Shannon’s index, evenness, and Faith’s PD. Differences were assessed based on Wilcoxon signed-rank test for each pair of indices. **C** Venn diagram illustrating shared versus unique ASVs in the phyllosphere and laimosphere. The number of corresponding ASVs is indicated, with corresponding percentages given in parentheses

The differences between phyllosphere and laimosphere were assessed more specifically by focusing on corresponding samples, i. e., pairs of phyllosphere and laimosphere samples that were collected from the same plant individual. PCA and PERMANOVA for this data subset confirmed the differences in the bacterial community composition of these two compartments (*R*^2^ = 0.108, *p* = 0.001) (Fig. 3A). As before, Shannon’s diversity index (*p* < 0.01) and evenness (*p* < 0.05) were higher in the laimosphere than the phyllosphere, while Faith’s PD remained at the same level (Fig. 3B). Despite equal Faith’s PD, we observed a higher overall ASV richness when looking at all laimosphere samples in comparison to the overall observed richness in all phyllosphere samples (Fig. 3C). In agreement with the higher overall richness, more unique ASVs (32% of total) were detected exclusively in the laimosphere than in the phyllosphere (24% of total) (Fig. 3C). This underlines that the above- and belowground parts of *T. landbeckii* plants provide distinct habitats for bacteria, with the laimosphere hosting a more even community with more unique ASVs than the phyllosphere.

Further habitat differentiation may exist between living and dead plants. To study this aspect, we had included some samples from dead plants, collected from the Salar Grande and Cerro Pajonal populations, where *T. landbeckii* populations were less vital compared to other study sites, with a remarkable number of dead plant individuals side by side to still living individuals (Fig. 1D). However, DNA extraction and PCR amplification of bacterial 16S rRNA genes from the dead plant material were of limited success. As a consequence, differences between living and dead plants could not be robustly assessed. They were overlaid by site-specific effects (Figure S2), which could not be excluded by data sub-setting due to the limited number of samples from dead plants per site. A further differentiation would have been expected, considering that plant-driven selection processes are relevant for shaping the phyllosphere microbiota. This requests further analyses in the future.

### Dominant bacterial classes and genera of *T. landbeckii* plants are well-known phyllosphere colonizers and include arthropod endosymbionts

**Fig. 4:**
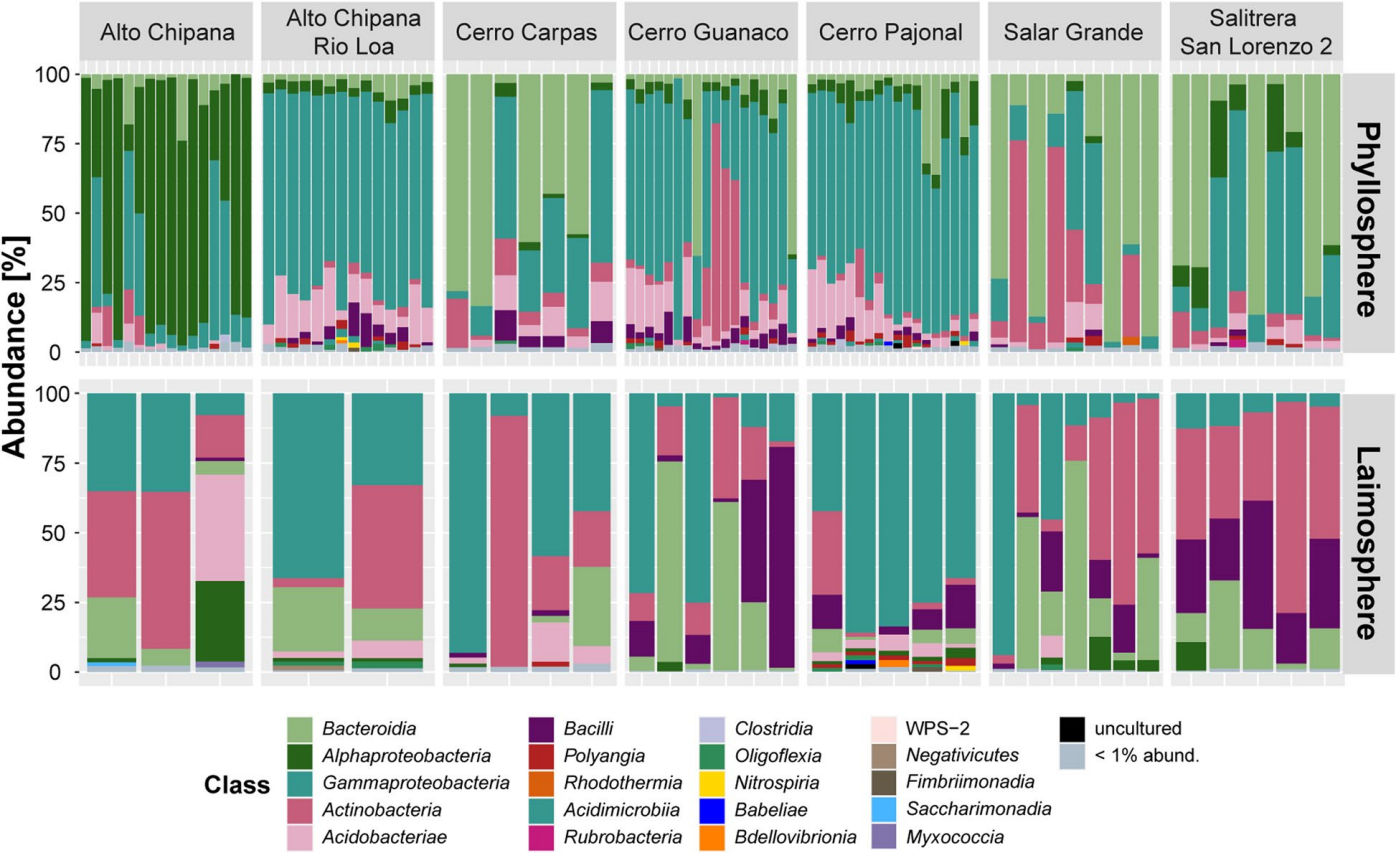
Bacterial community composition in phyllosphere and laimosphere samples, collected at seven different study sites. All samples collected per site are included here

Phyllosphere bacterial communities of the individual plants were dominated by *Alphaproteobacteria*, *Gammaproteobacteria*, *Actinobacteria*, or *Bacteroidia* (Fig. 4), which are known to represent dominant classes in the phyllosphere [14]. Likewise, several of the dominant genera (> 10% relative abundance in a phyllosphere sample; Figure S3) are well-known phyllosphere colonizers. This applies to all dominant *Gammaproteobacteria*, i.e., *Pseudomonas*, *Massilia*, and *Ralstonia*, as well as *Hymenobacter* (class *Bacteroidia*), which have all been reported to occur prominently or as part of a phyllosphere core microbiota on other plants (e.g., [14, 51, 60–66]). Similarly, dominant genera within the class *Actinobacteria*, i.e., *Modestobacter* and *Kineococcus*, are known as phyllosphere colonizers [51, 61]. Several type strains of these two actinobacterial genera were isolated from halophytic plants [67–70], desert [71, 72], or saline environments [73]. Thus, members of these genera may be particularly well adapted to water-limiting conditions, likewise as reported for soil-dwelling *Actinobacteria* [74, 75]. However, we did not observe a general dominance of *Actinobacteria* in the phyllosphere, as often seen in hyperarid desert soils [4, 7, 8]. Instead, a predominance of *Actinobacteria* was only seen on some plant individuals from two study sites, Cerro Guanaco and Salar Grande (Fig. 4), indicating that drought-adapted members of this genus are not consistently dominating in the phyllosphere.

Similarly, the genera *Wolbachia* and “*Candidatus* Uzinura” occurred dominantly in the phyllosphere of some *T. landbeckii* individuals. These organisms are known as endosymbionts from plant(-sap) feeding arthropods and may use plants as intermediate hosts, being possibly transferred to plant tissue upon insect feeding [60, 76–78]. This is supported by a rather inconsistent occurrence of these taxa on plant individuals within and between the study sites, which is a logical consequence of selective (e.g., related to plant fitness) or stochastic insect attacks of plant individuals. The detection of these two endosymbionts, likewise as of endosymbionts of the family *Morganellaceae *(Figure S3), raises the question of which arthropods might live in these hyperarid environments. During field sampling, no insect infestation of the *Tillandsia* plants was evident, but the presence of the endophytes suggests that the plants support also insect life under hyperarid conditions.

### Characteristic and unusual colonizers in the phyllosphere of *T. landbeckii* plants in comparison to the laimosphere

**Fig. 5:**
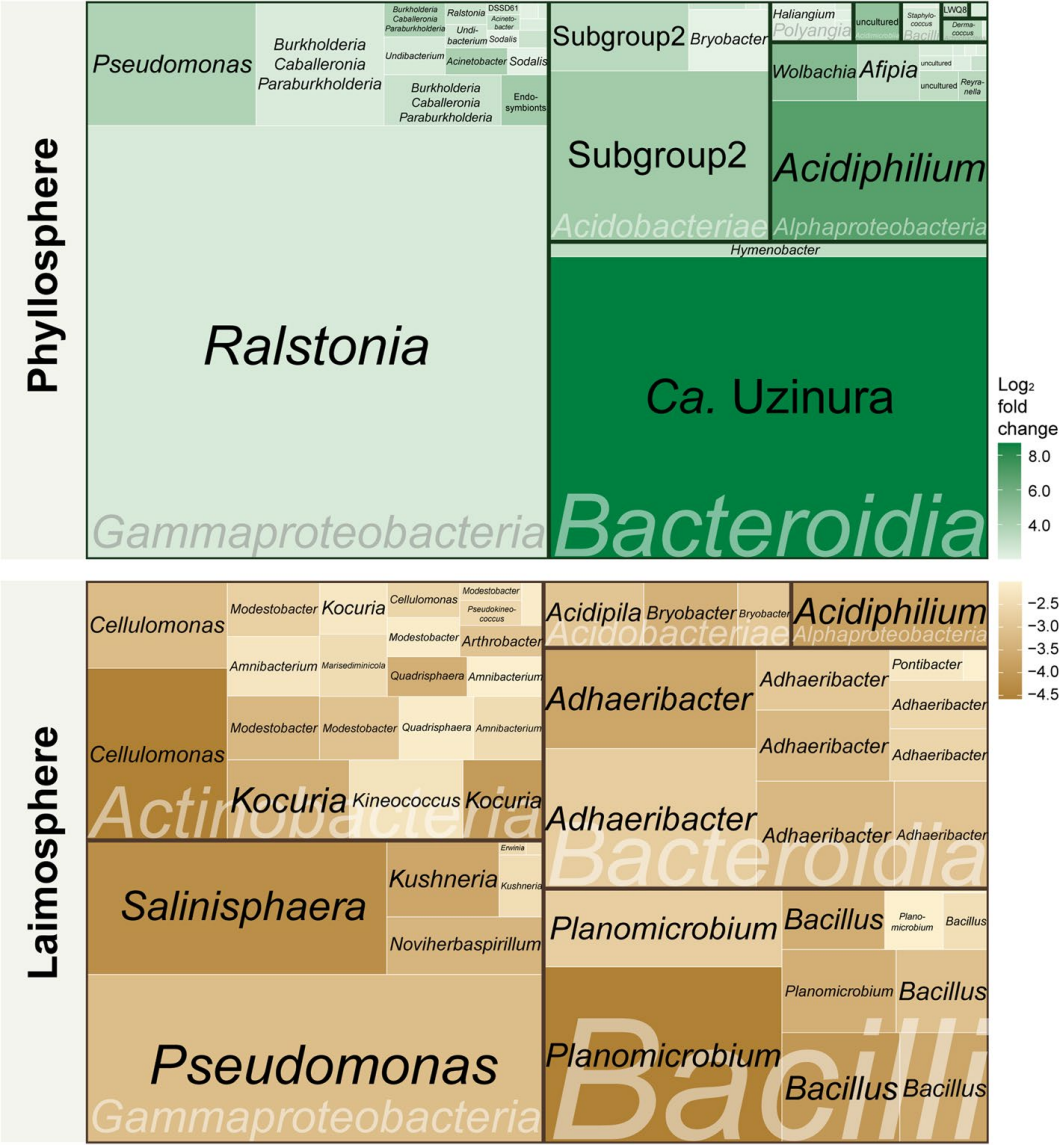
Significantly enriched ASVs in the phyllosphere and laimosphere of *T. landbeckii* plants identified by ANCOM-BC. Displayed are ASVs with *p*_*adj*_*.* ≤ 0.05 and log_2_ fold changes of  ≥ 2 (abundant in phyllosphere) and ≤ −2 (abundant in laimosphere), with the color gradient illustrating the fold change according to the legend. Each light-bordered box represents one ASV, with the genus name given in black. The class names are indicated in light color, grouping the ASVs in big boxes with dark borders. The segment sizes reflect the mean relative abundance of the ASVs across all samples from the respective compartment, as included in ANCOM-BC, which included all plants from which a phyllosphere and corresponding laimosphere sample was taken. The names of the low-abundant taxa can be found in Table S3

To identify phyllosphere-specific ASVs more systematically in comparison to the laimosphere, differential abundance analysis was performed using ANCOM-BC on the reduced dataset of corresponding phyllosphere-laimosphere samples obtained from 27 plant individuals across the seven study sites. In the phyllosphere, 51 ASVs were identified as significantly enriched with *p*_*adj*_. ≤ 0.05 and log_2_ fold change ≥ 2 (Fig. 5 and Table S3). These ASVs represented in total 85% of the mean relative abundance in the phyllosphere samples, but only 24% of the mean relative abundance in the laimosphere samples (Table S3), indicating that several of the phyllosphere-enriched ASVs were dominant members in this community and clearly depleted in the laimosphere. They represented mostly *Gammaproteobacteria *(18 ASVs, summed mean relative abundance 42.0%), followed by *Alphaproteobacteria* (13 ASVs, 7.1%), *Acidobacteriae *(6 ASVs, 8.7%), and *Bacteroidia *(2 ASVs, 42.1%).

The most abundant ASV (ASV406, 32.9% mean relative abundance) in the phyllosphere was representing *Ralstonia* and had a log_2_ fold change of 2.5 in comparison to the laimosphere. *Ralstonia* has been reported as dominant or consistent phyllosphere colonizer in some other studies [60, 63, 79]. The genus contains some serious soil-born plant pathogens (*Ralstonia solanacearum*, *Ralstonia syzygii*, *Ralstonia pseudosolanacearum*) causing bacterial wilt [80], but is not yet known as pathogen of Bromeliaceae. Considering that plants appeared healthy, these were probably nonpathogenic strains, or the plants are not susceptible. The ASV showing the strongest enrichment (log_2_ fold change 8.7) was “*Candidatus* Uzinura” (ASV280, with 21.7% mean relative abundance, Table S3), the endosymbiont of arthropods discussed above. Further ASVs occurring with high relative abundance and enriched in the phyllosphere (log_2_ fold change between 2.4 and 6.8) were members of *Acidiphilium* (ASV43), *Wolbachia* (ASV89), *Pseudomonas* (ASV108), *Burkholderia-Caballeronia-Paraburkholderia* (ASV334), and subgroup2 of the *Acidobacteriae *(ASV316) (Fig. 5 and Table S3). Several of these and further less-abundant, significantly enriched taxa such as *Acinetobacter*, *Sphingomonas*, *Mesorhizobium*, and *Rhodococcus* (Fig. 5) are well-known phyllosphere colonizers [14], thus underlining that *T. landbeckii* provides a characteristic phyllosphere habitat. The presence of these taxa may be explained by the fact that the phyllosphere of many plants, not only desert plants, is representing a rather hostile environment where microorganisms are exposed to drought and UV radiation [14]. Phyllosphere colonizers are thus somewhat adapted to these conditions, possibly enabling some taxa to colonize even *T. landbeckii* plants. Besides, it has to be kept in mind that we did not separate endophytes from epiphytes in this study. Endophytes may need less adaptations to the harsh conditions compared to epiphytes for survival in association with plants growing in hyperarid environments.

The enrichment of two ASVs representing *Acidobacteriae* subgroup2 in the phyllosphere was rather exceptional. *Acidobacteriae* have been detected in the phyllosphere, especially of tropical trees, but predominantly subgroup1, not subgroup2 [81–83]. Similarly, the presence of *Acidiphilium* was rather unexpected, as it is usually occurring in acidic, mineral, and oligotrophic environments [84], but not in the phyllosphere. This taxon was primarily detected at Alto Chipana but consistently present with lower relative abundance in the phyllosphere of all other plant individuals. Due to the consistent occurrence, these otherwise fairly uncommon phyllosphere colonizers appear to be part of a *T. landbeckii*-specific microbiota, therewith contributing to the plant-host specificity of the microbiome, which is well-known to exist between different taxonomic groups of plants, sometimes even down to plant cultivar level [85–87]. Their occurrence may result from the extreme conditions the plants and associated bacteria are exposed to in hyperarid environments. However, microbiota studies from plants growing in semiarid or arid deserts do not provide further evidence for this; the occurrence of these genera has not been reported there [18–20]. It is thus tempting to speculate that the transition from arid to hyperarid conditions introduces specific changes in the plant-associated microbiota, analogous to observations made in soil [4], or that *T. landbeckii* plants have some unique features that support these taxa, especially in the phyllosphere.

Taken together, these results suggest that the *T. landbeckii* phyllosphere bacterial community hosts several taxa commonly known from plants grown under less arid conditions but includes in addition some particular taxa. The detected actinobacterial genera and *Bacilli* suggest that a selection of particularly drought-tolerant taxa may occur. The relevance of drought adaptation of the *T. landbeckii* microbiota should be studied in more detail in the future, not only for the *Actinobacteria *and *Bacilli* but also for the well-known phyllosphere colonizers. Likewise, the plant traits and processes leading to the specific presence of subgroup2 *Acidobacteriae* and *Acidiphilium* deserve further attention.

### The *T. landbeckii* laimosphere hosts soil and rhizosphere-dwelling bacteria in addition to phyllosphere colonizers

Similar to the phyllosphere, *Gammaproteobacteria*, *Actinobacteria*, or *Bacteroidia *dominated in the laimosphere (Fig. 4). In addition, *Bacilli* were quite prominent here, while *Alphaproteobacteria* were less abundant. Focusing on dominant genera (> 10% relative abundance in a sample), 11 of the 17 genera that were dominant in the phyllosphere were also found as dominant taxa in the laimosphere (Figure S3). When evaluating all ASVs, the overlap between the phyllosphere and laimosphere was 44% (Fig. 3C). This indicates that the laimosphere shares a substantial part of its microbiota with the phyllosphere. Besides, the laimosphere hosted several additional abundant genera compared to the phyllosphere (Figure S3). These were primarily members of the *Actinobacteria*, *Bacteroidia*, and *Gammaproteobacteria* (Figs. 4 and S3). Moreover, some dominant members of the phylum *Acidobacteriota* were present. In agreement with the higher number of dominant genera, 32% of the ASVs were uniquely detected in these samples (Fig. 3C). The specific comparison between phyllosphere and laimosphere by ANCOM-BC revealed 52 ASVs to be significantly enriched with a log_2_ fold change ≤ −2 in the laimosphere (*p*_*adj.*_ ≤ 0.05) relative to the phyllosphere. In total, these 52 ASVs represented 44% of the mean relative abundance of the bacterial community in the laimosphere samples but less than 1% of the mean relative abundance of the phyllosphere samples (Fig. 5 and Table S3), therewith underlining the distinctiveness of the two compartments. Most of these ASVs belonged to the class *Actinobacteria* (24 ASVs, 11.3% summed mean relative abundance), followed by *Bacilli* (10 ASVs, 9.5%), *Bacteroidia* (10 ASVs, 9.0%), and *Gammaproteobacteria *(7 ASVs, 11.6%).

The most prominent ASV with significant enrichment in the laimosphere was a member of the genus *Pseudomonas* (ASV81, 6.5% mean relative abundance, log_2_ fold change of −3.1), which was different from the abundant *Pseudomonas* ASV in the phyllosphere (ASV108, 3.4%, log_2_ fold change of 3.8, Table S3). Likewise, different ASVs representing *Acidiphilium* and *Bryobacter* were enriched in the phyllosphere and laimosphere (Fig. 5). This points to niche differentiation at species or even strain level within these genera. Among the most strongly enriched taxa in the laimosphere (log_2_ fold change between −3.5 and −4.6) were *Cellulomonas*, *Kocuria*, *Modestobacter* (all from the class Actinobacteria), *Planomicrobium* and *Bacillus* (*Bacilli*), *Salinisphaera*, *Kushneria* and *Noviherbaspirillum* (*Gammaproteobacteria*), and *Adhaeribacter* (*Bacteroidia*), which were mostly represented by several differentially abundant ASVs (Table S3). All these genera include soil-dwelling organisms, and most include strains that have been reported to exist under dry or high-salt conditions [88–92]. Moreover, some of those were also detected in our surface soil samples (Table S2), indicating that those could be soilborne taxa, which colonized the buried plant parts in addition. In further agreement with this assumption, several of these taxa (*Cellulomonas*, *Kocuria*, *Planomicrobium*, *Bacillus*, *Pseudomonas*, and *Kushneria*) have been reported to establish in the rhizosphere of desert plants or halophilic plants and have potentially plant beneficial traits [88, 93–97]. Furthermore, some have the capability to degrade plant residue (*Cellulomonas*, *Bacillus*, *Adhaeribacter*, *Pseudomonas*) [98–102]. This indicates that the laimosphere is colonized in part by soil-derived bacteria, which probably benefit from plant carbon. However, it is not yet known whether organic carbon is leaking from the buried plant shoots or whether the bacteria actively decompose the buried shoots under conditions when sufficient water is available for metabolic activity. Most of the time, soil water contents will be too low to allow decay of these buried shoots [41], therewith guaranteeing the maintenance of the anchorage function of these shoots upon burial. These remain largely intact, sometimes for centuries even after burial of the complete plant [38, 103], due to very slow degradation processes. Upon burial, the microbiota probably undergoes a very slow transition from a typical phyllosphere microbiota towards a more saprotrophic microbiota.

### Contrasting biogeographic patterns exist in the phyllosphere and laimosphere of *T. landbeckii*

**Fig. 6:**
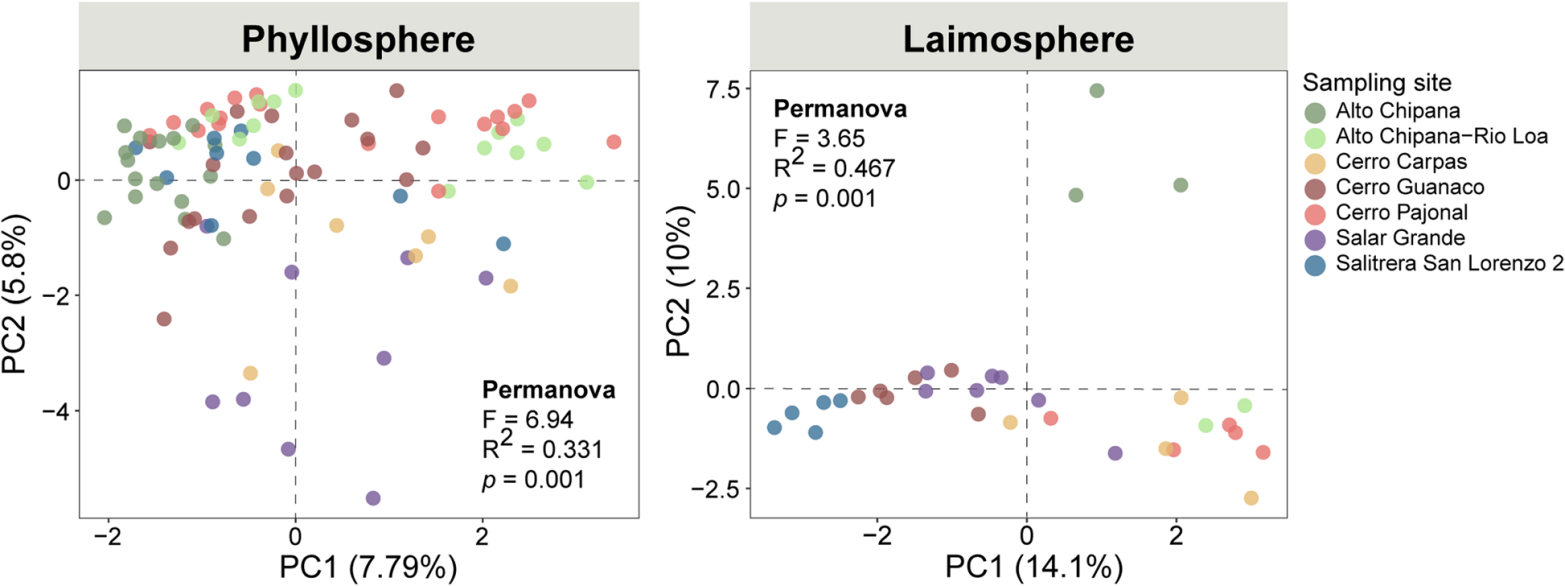
Differences in bacterial community composition in the phyllosphere and laimosphere related to sampling site. Shown are principal component plots and PERMANOVA results based on robust Aitchison distance matrices

Biogeographic patterns are commonly observed in the plant-associated microbiota [16, 85]. To gain insight into spatial community heterogeneity of the plant-associated microbiota of *T. landbeckii* in the two compartments, the data were subsetted by compartment, and the bacterial community structure was compared between study sites. All three alpha-diversity indices, i.e., Shannon’s index, evenness, and Faith’s PD, differed for the phyllosphere bacterial communities between the different study sites (Figure S4). For the laimosphere, differences were less evident. Regarding beta diversity, PCA and PERMANOVA revealed spatial variation in the phyllosphere (*R*^2^ = 0.331, *p* < 0.001), which was even more pronounced in the laimosphere (*R*^2^ = 0.467, *p* < 0.001) (Fig. 6). In the laimosphere, samples from Alto Chipana appeared most separated in the PCA plot, while this was not evident in the phyllosphere, where Salar Grande samples appeared most distinct (Fig. 6). This underlines that the two plant-associated bacterial communities are controlled by different environmental factors. Distance decay will contribute to spatial patterns, but this was not the main factor for differences in the two compartments of *T. landbeckii* in the Atacama Desert, as sample clustering by sites in the PCA plots does not reflect geographic distances between the study sites. Consequently, other site-specific environmental factors must be more relevant drivers for community assembly. Assuming that the laimosphere is influenced by the surrounding soil substrate, we evaluated comparatively the biogeographic patterns of the bacterial communities in the surface soil samples, revealing that strong spatial patterns exist also in these samples (*R*^2^ = 0.517, *p* < 0.001), with samples from Alto Chipana and Salitrera San Lorenzo 2 being most distinct from the other samples (Figure S1). As samples from Alto Chipana were also most distinct in the laimosphere from all others and those from Salitrera San Lorenzo 2 were separated from many others along the first axis (Fig. 6), soil-related traits and the recruitment of soil-derived microorganisms in the laimosphere likely contributed to the variation between sites in the laimosphere. Soil traits that may play a role are pH, organic carbon content, electric conductivity, or the concentration of specific salts [4, 104, 105]. The plant itself is probably another factor, being relevant for both the phyllosphere and laimosphere. The studied *T. landbeckii* plants are known to be genetically distinct [31], resulting in potential phenotypic differences, which might lead to altered community structures [106–109]. This also deserves a more detailed evaluation in the future. Moreover, climatic conditions, such as fog availability, may have an influence in particular on the phyllosphere microbiota, acting either directly or indirectly via altered plant physiology.

### Temporal changes provide evidence for environmentally responsive bacterial communities in the phyllosphere of *T. landbeckii*

**Fig. 7:**
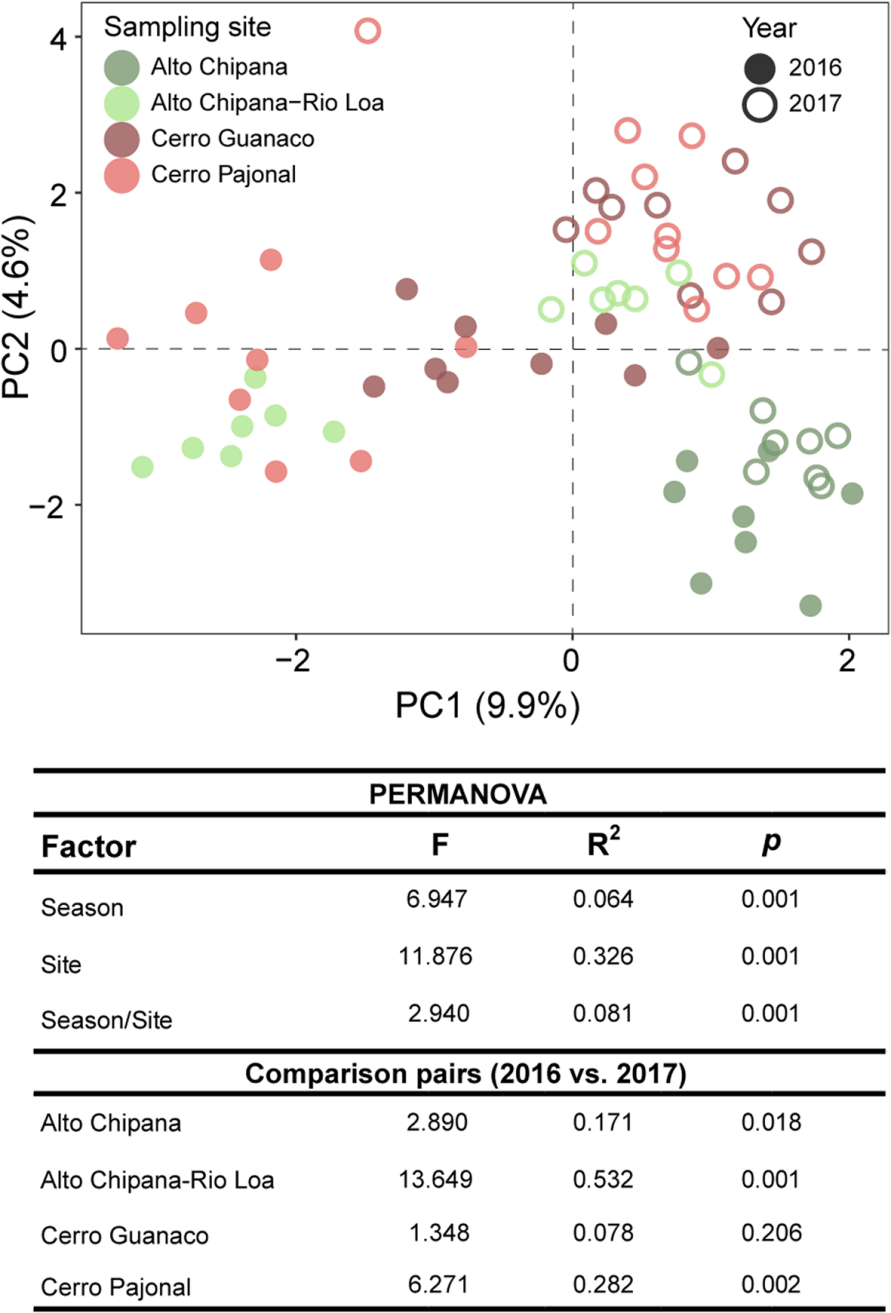
Differences in bacterial community composition in the phyllosphere related to sampling site and season. Shown are principal component plots and PERMANOVA results based on robust Aitchison distance matrices

Having proven the presence of specific bacterial communities in the different compartments of *T. landbeckii* and the existence of spatial patterns, we were interested in the environmental responsiveness of the plant-associated microbiota. Due to the limitation of water, microbial metabolic activity and, therewith, responsiveness might be strongly limited, similarly as known from the hyperarid desert soil in the absence of water [6]. To gain evidence, we analyzed the temporal dynamics of the bacterial communities in the phyllosphere. Therefore, we collected samples from four study sites twice, in spring (October 2016), and half a year later in autumn 2017 (March). Overall, all three alpha-diversity indices changed significantly over time (*p* ≤ 0.002) (Figure S5). A more detailed analysis within each site revealed that such changes were not observed consistently across all sites. A significant decline in bacterial diversity (Shannon’s index and Faith’s PD) was observed at Cerro Pajonal, whereas a less pronounced decline was seen in samples from Alto Chipana-Rio Loa (Faith’s PD only). PCA revealed clear differences related to season in beta diversity, partly reflected by a low though significant *R*^2^ value in PERMANOVA (*R*^2^ = 0.064, *p* = 0.001) (Fig. 7). The season-related changes were superimposed by the stronger spatial patterning (*R*^2^ = 0.326, *p* = 0.001) and a combined season/site effect (*R*^2^ = 0.081, *p* = 0.001). When assessing the temporal changes site specifically, significant changes in the phyllosphere bacterial community composition over time were seen at three study sites (PERMANOVA *R*^2^ ranging from 0.171 to 0.532) (Fig. 7). This leads to the conclusion that the phyllosphere microbiota does undergo temporal changes. It appears likely that these changes are related to weather conditions, which are known to be a controlling factor for plant-associated microorganisms in general [110, 111]. Water availability via incoming fog may be a major factor here. Fog availability is higher between July and November than in the other months [41, 112, 113] and was therewith higher at the first sampling event than the second. In this context, the observed decline in alpha diversity appears reasonable, assuming that only the better adapted microorganisms and endophytes withstand the dryer period of the year. Besides a direct impact of fog on the microorganisms, the increased availability of fog-derived water has implications on plant productivity. *T. landbeckii* plants at Salitrera San Lorenzo 2 were reported to have lowest growth rates in May to August and highest rates in August to November, along with the higher fog availability at this time [113]. This may change habitat conditions for the phyllosphere microorganisms, contributing to seasonal community compositional changes. The specific roles of fog and host plant factors request further evaluation in the future. The main conclusion drawn from the observed differences here is the existence of temporal variation, serving as indicator for responsiveness of the *T. landbeckii* phyllosphere microbiota to environmental cues.

## Conclusion

In this study, we demonstrated that *T. landbeckii* plants, which grow under extreme conditions in hyperarid deserts, provide distinct habitats for microorganisms. The phyllosphere hosts in part well-known phyllosphere colonizers, indicating that *T. landbeckii* plants provide a refugium of microbial life in hyperarid deserts. In addition, some detected genera are rather unique for the *Tillandsia* phyllosphere, pointing to a selection of particular host- or habitat-specific taxa. The bacterial community of the laimosphere was observed to be partially distinct from the one in the phyllosphere, in part due to the presence of soil-dwelling taxa. Biogeographic patterns were more prominent in the laimosphere than the phyllosphere, indicating that the community assembly in these two compartments is driven by different deterministic as well as stochastic processes. Climate, especially fog and therewith water availability, appears to be an important factor acting in particular on the phyllosphere microbiota, either directly or indirectly via plant fitness, whereas soil traits such as water storage capacity may modulate the laimosphere microbiota in addition. In particular, the role of fog for the plant-associated microbiota deserves more attention in future studies, as it may explain the temporal variation observed in this study. Both the spatial and even more so the temporal variation in the bacterial community structure, we observed here points to a microbiota that is responsive to environmental cues. Thus, it appears unlikely that the plant-associated microbiota of *T. landbeckii*, growing under hyperarid conditions, is merely the result of an accumulation of (dead) microorganisms from dust depositional processes. Instead, the plant provides specific species-rich refugia of life for microorganisms above- and belowground in this hostile hyperarid desert. The microorganisms that colonize *T. landbeckii* should be studied in more detail with regard to their in situ metabolic activity, their adaptation mechanisms to live in this hyperarid environment, and how these differ from those of microorganisms living in the phyllosphere under less arid conditions. Moreover, the possible role of the *T. landbeckii* microbiome for plant growth support deserves attention.

### Supplementary Information

**Additional file 1: Supplementray figures: Fig. S1.** Principal component plot of surface soil samples showing the variation in bacterial community composition between samples from different study sites. DNA extraction negative control (EC) and PCR negative control (EC) are included. They both remained slightly positive after applying a decontamination algorithm and cluster with surface soil samples from some, though not all sampling sites. **Fig. S2.** Principal component plots showing the variation in bacterial community composition in phyllosphere and laimosphere samples from living and dead plant material, collected at Salar Grande and Cerro Pajonal. The tables below the figures summarize PERMANOVA results. **Fig. S3.** Heat trees showing the bacterial community structure up to genus level in the complete datasets of the phyllosphere and laimosphere. The color and size of nodes and edges are correlated with the sum of the mean abundance and number of ASVs, respectively. The most abundant genera (> 10% abundance in individual samples) are highlighted. The abundance value of 0.001 was set as a threshold to remove low-abundance taxa and reduce the complexity of the plots. **Fig. S4.** Bacterial diversity in the phyllosphere and laimosphere of *T. landbeckii*. Shown are Shannon’s index (upper row), evenness (middle row) and Faith’s PD (lower row). Significant differences in dependence on study site were evaluated based on Kruskal-Wallis tests. Please note the different y-axis scales for phyllosphere and laimosphere. **Fig. S5.** Bacterial diversity in the phyllosphere in dependence on the season for the samples from four different study sites. Shown are Shannon’s index, evenness, and Faith’s PD. Significant differences in dependence on the season were evaluated based on Kruskal-Wallis tests and are reported above the respective panel. Pairwise comparisons within each study site were done using Wilcoxon signed rank exact test for the paired samples and the Wilcoxon sum exact test for non-paired samples (e.g. Cerro Pajonal 2016 vs. 2017). Significance values: *p < 0.05, ***p < 0.001.

**Additional file 2: Supplementary tables: Table S1.** Information about sample number and sequencing results. ** Table S2.** The complete ASV table with raw read numbers and taxonomic ranking after decontamination. **Table S3.** The differentially enriched ASVs in phyllosphere and laimosphere assessed by ANCOM-BC analysis.

## Data Availability

The 16S rRNA gene sequence reads generated in this study have been deposited in the European Nucleotide Archive (ENA) under accession no. PRJEB57843.

## References

[1] Warren-Rhodes KA, Rhodes KL, Pointing SB, Ewing SA, Lacap DC, Gómez-Silva B (2006). Hypolithic cyanobacteria, dry limit of photosynthesis, and microbial ecology in the hyperarid Atacama Desert. Microb Ecol.

[2] Azua-Bustos A, González-Silva C, Fairén AG (2022). The Atacama Desert in northern Chile as an analog model of Mars. Front Astron Space Sci.

[3] Bahram M, Hildebrand F, Forslund SK, Anderson JL, Soudzilovskaia NA, Bodegom PM (2018). Structure and function of the global topsoil microbiome. Nature.

[4] Knief C, Bol R, Amelung W, Kusch S, Frindte K, Eckmeier E (2020). Tracing elevational changes in microbial life and organic carbon sources in soils of the Atacama Desert. Glob Planet Change.

[5] Navarro-González R, Rainey FA, Molina P, Bagaley DR, Hollen BJ, de la Rosa J (2003). Mars-like soils in the Atacama Desert, Chile, and the dry limit of microbial life. Science.

[6] Schulze-Makuch D, Wagner D, Kounaves SP, Mangelsdorf K, Devine KG, de Vera J-P (2018). Transitory microbial habitat in the hyperarid Atacama Desert. Proc Natl Acad Sci USA.

[7] Vasquez-Dean J, Maza F, Morel I, Pulgar R, Gonzalez M (2020). Microbial communities from arid environments on a global scale. A systematic review. Biol Res.

[8] Leung PM, Bay SK, Meier DV, Chiri E, Cowan DA, Gillor O (2020). Energetic basis of microbial growth and persistence in desert ecosystems. mSystems.

[9] Pan H, Gao H, Peng Z, Chen B, Chen S, Liu Y (2022). Aridity threshold induces abrupt change of soil abundant and rare bacterial biogeography in dryland ecosystems. mSystems.

[10] Azúa-Bustos A, González-Silva C, Mancilla RA, Salas L, Gómez-Silva B, McKay CP (2011). Hypolithic cyanobacteria supported mainly by fog in the coastal range of the Atacama Desert. Microb Ecol.

[11] DiRuggiero J, Wierzchos J, Robinson CK, Souterre T, Ravel J, Artieda O (2013). Microbial colonisation of chasmoendolithic habitats in the hyper-arid zone of the Atacama Desert. Biogeosciences.

[12] Robinson CK, Wierzchos J, Black C, Crits-Christoph A, Ma B, Ravel J (2015). Microbial diversity and the presence of algae in halite endolithic communities are correlated to atmospheric moisture in the hyper-arid zone of the Atacama Desert. Environ Microbiol.

[13] Bulgarelli D, Schlaeppi K, Spaepen S, van Ver Loren Themaat E, Schulze-Lefert P (2013). Structure and functions of the bacterial microbiota of plants. Annu Rev Plant Biol.

[14] Vorholt JA (2012). Microbial life in the phyllosphere. Nat Rev Microbiol.

[15] Al Ashhab A, Meshner S, Alexander-Shani R, Dimerets H, Brandwein M, Bar-Lavan Y (2021). Temporal and spatial changes in phyllosphere microbiome of acacia trees growing in arid environments. Front Microbiol.

[16] Coleman-Derr D, Desgarennes D, Fonseca-Garcia C, Gross S, Clingenpeel S, Woyke T (2016). Plant compartment and biogeography affect microbiome composition in cultivated and native *Agave* species. New Phytol.

[17] Finkel OM, Burch AY, Elad T, Huse SM, Lindow SE, Post AF (2012). Distance-decay relationships partially determine diversity patterns of phyllosphere bacteria on *Tamrix* trees across the Sonoran Desert. Appl Environ Microbiol.

[18] Fonseca-García C, Coleman-Derr D, Garrido E, Visel A, Tringe SG, Partida-Martínez LP (2016). The cacti microbiome: interplay between habitat-filtering and host-specificity. Front Microbiol.

[19] Qvit-Raz N, Finkel OM, Al-Deeb TM, Malkawi HI, Hindiyeh MY, Jurkevitch E (2012). Biogeographical diversity of leaf-associated microbial communities from salt-secreting *Tamarix* trees of the Dead Sea region. Res Microbiol.

[20] Tahtamouni ME, Khresat SE, Lucero M, Sigala J, Unc A (2016). Diversity of endophytes across the soil-plant continuum for *Atriplex* spp. in arid environments. J Arid Land.

[21] Araya JP, González M, Cardinale M, Schnell S, Stoll A (2020). Microbiome dynamics associated with the Atacama flowering Desert. Front Microbiol.

[22] Eshel G, Araus V, Undurraga S, Soto DC, Moraga C, Montecinos A (2021). Plant ecological genomics at the limits of life in the Atacama Desert. Proc Natl Acad Sci USA.

[23] Lopez BR, Bashan Y, Bacilio M (2011). Endophytic bacteria of *Mammillaria fraileana*, an endemic rock-colonizing cactus of the southern Sonoran Desert. Arch Microbiol.

[24] Maldonado JE, Gaete A, Mandakovic D, Aguado-Norese C, Aguilar M, Gutiérrez RA (2022). Partners to survive: *Hoffmannseggia doellii* root-associated microbiome at the Atacama Desert. New Phytol.

[25] Dunai TJ, Melles M, Quandt D, Knief C, Amelung W (2020). Whitepaper: Earth – evolution at the dry limit. Glob Planet Change.

[26] Ruhm J, Böhnert T, Weigend M, Merklinger FF, Stoll A, Quandt D (2020). Plant life at the dry limit - spatial patterns of floristic diversity and composition around the hyperarid core of the Atacama Desert. PLoS ONE.

[27] Rundel PW, Dillon MO, Palma B, Mooney HA, Gulmon S, Ehleringer J (1991). The phytogeography and ecology of the coastal Atacama and Peruvian deserts. Aliso.

[28] Pinto R, Barría I, Marquet PA (2006). Geographical distribution of *Tillandsia* lomas in the Atacama Desert, northern Chile. J Arid Environ.

[29] Hesse R (2012). Spatial distribution of and topographic controls on *Tillandsia* fog vegetation in coastal southern Peru: remote sensing and modelling. J Arid Environ.

[30] Wolf N, Siegmund A, del Río C, Osses P, García JL. Remote sensing-based detection and spatial pattern analysis for geo-ecological niche modeling of *Tillandsia* spp. in the Atacama, Chile. Int Arch PhotoGramm Remote Sens Spatial Inf Sci. 2016;41:251. 10.5194/isprs-archives-XLI-B2-251-2016.

[31] Merklinger FF, Zheng Y, Luebert F, Harpke D, Böhnert T, Stoll A (2020). Population genomics of *Tillandsia landbeckii* reveals unbalanced genetic diversity and founder effects in the Atacama Desert. Glob Planet Change.

[32] Houston J (2006). Variability of precipitation in the Atacama Desert: its causes and hydrological impact. Int J Climatol.

[33] Rundel PW, Dillon MO (1998). Ecological patterns in the *Bromeliaceae* of the lomas formations of coastal Chile and Peru. Plant Syst Evol.

[34] Rundel PW, Palma B, Dillon MO, Sharifi MR, Nilsen ET, Boonpragob K (1997). *Tillandsia landbeckii* in the coastal Atacama Desert of northern Chile. Rev Chil de Hist Nat.

[35] Belmonte E, Arriaza B, Arismendi M, Sepúlveda G. Foliar anatomy of three native species of *Tillandsia* L. from the Atacama Desert, Chile. Plants. 2022;11:870. 10.3390/plants11070870. 10.3390/plants11070870 PMC9003348 35406850

[36] Raux PS, Gravelle S, Dumais J (2020). Design of a unidirectional water valve in *Tillandsia*. Nat Commun.

[37] González AL, Fariña JM, Pinto R, Pérez C, Weathers KC, Armesto JJ (2011). Bromeliad growth and stoichiometry: responses to atmospheric nutrient supply in fog-dependent ecosystems of the hyper-arid Atacama Desert. Chile Oecologia.

[38] Jaeschke A, Böhm C, Merklinger FF, Bernasconi SM, Reyers M, Kusch S (2019). Variation in δ^15^N of fog-dependent *Tillandsia* ecosystems reflect water availability across climate gradients in the hyperarid Atacama Desert. Glob Planet Change.

[39] Magyarosy A, Hancock JG (1972). Microbial population of the laimosphere of squash (*Cucurbita maxima*). Plant Soil.

[40] Alfaro FD, Manzano M, Almiray C, García J-L, Osses P, del Rio C (2021). Soil bacterial community structure of fog-dependent *Tillandsia landbeckii* dunes in the Atacama Desert. Plant Syst Evol.

[41] Moat J, Orellana-Garcia A, Tovar C, Arakaki M, Arana C, Cano A (2021). Seeing through the clouds – mapping desert fog oasis ecosystems using 20 years of MODIS imagery over Peru and Chile. Int J Appl Earth Obs Geoinf.

[42] Becker MF, Hellmann M, Knief C (2022). Spatio-temporal variation in the root-associated microbiota of orchard-grown apple trees. Environ Microbiome.

[43] Ikenaga M, Sakai M (2014). Application of locked nucleic acid (LNA) oligonucleotide–PCR clamping technique to selectively PCR amplify the SSU rRNA genes of bacteria in investigating the plant-associated community structures. Microbes Environ.

[44] Bolyen E, Rideout JR, Dillon MR, Bokulich NA, Abnet CC, Al-Ghalith GA (2019). Reproducible, interactive, scalable and extensible microbiome data science using QIIME 2. Nat Biotechnol.

[45] Callahan BJ, McMurdie PJ, Rosen MJ, Han AW, Johnson AJA, Holmes SP (2016). DADA2: high-resolution sample inference from Illumina amplicon data. Nat Methods.

[46] Quast C, Pruesse E, Yilmaz P, Gerken J, Schweer T, Yarza P (2013). The SILVA ribosomal RNA gene database project: improved data processing and web-based tools. Nucleic Acids Res.

[47] Yilmaz P, Parfrey LW, Yarza P, Gerken J, Pruesse E, Quast C (2013). The SILVA and “All-species Living Tree Project (LTP)” taxonomic frameworks. Nucleic Acids Res.

[48] Pruesse E, Peplies J, Glöckner FO (2012). SINA: accurate high-throughput multiple sequence alignment of ribosomal RNA genes. Bioinformatics.

[49] McMurdie PJ, Holmes S (2013). phyloseq: an R package for reproducible interactive analysis and graphics of microbiome census data. PLoS ONE.

[50] Davis NM, Proctor DM, Holmes SP, Relman DA, Callahan BJ (2018). Simple statistical identification and removal of contaminant sequences in marker-gene and metagenomics data. Microbiome.

[51] Sahu KP, Kumar A, Sakthivel K, Reddy B, Kumar M, Patel A (2022). Deciphering core phyllomicrobiome assemblage on rice genotypes grown in contrasting agroclimatic zones: implications for phyllomicrobiome engineering against blast disease. Environ Microbiome.

[52] Martino C, Morton JT, Marotz CA, Thompson LR, Tripathi A, Knight R (2019). A novel sparse compositional technique reveals microbial perturbations. mSystems.

[53] Oksanen J, Simpson G, Blanchet F, Kindt R, Legendre P, Minchin P, et al. Vegan: community ecology package - R package version 2.6–2. 2022. https://github.com/vegandevs/vegan.

[54] Martinez AP. pairwiseAdonis: pairwise multilevel comparison using Adonis - R package version 0.4. 2017. https://github.com/pmartinezarbizu/pairwiseAdonis.

[55] Lin H, Peddada SD (2020). Analysis of compositions of microbiomes with bias correction. Nat Commun.

[56] Wickham H (2016). ggplot2: elegant graphics for data analysis.

[57] Russel J. MicEco: various functions for microbial community data - R package version 0.9.18. 2022. https://github.com/Russel88/MicEco.

[58] Foster ZSL, Sharpton TJ, Grünwald NJ (2017). Metacoder: an R package for visualization and manipulation of community taxonomic diversity data. PLoS Comput Biol.

[59] Delmotte N, Knief C, Chaffron S, Innerebner G, Roschitzki B, Schlapbach R (2009). Community proteogenomics reveals insights into the physiology of phyllosphere bacteria. Proc Natl Acad Sci USA.

[60] Chun S-J, Cui Y, Yoo S-H, Lee JR (2022). Organic connection of holobiont components and the essential roles of core microbes in the holobiont formation of feral *Brassica napus*. Front Microbiol.

[61] Durand A, Maillard F, Alvarez-Lopez V, Guinchard S, Bertheau C, Valot B (2018). Bacterial diversity associated with poplar trees grown on a Hg-contaminated site: community characterization and isolation of Hg-resistant plant growth-promoting bacteria. Sci Total Environ.

[62] Janakiev T, Berić T, Stević T, Stanković S, Bačić J, Majstorović H (2022). The microbiome of the ‘Williams’ pear variety grown in the organic orchard and antifungal activity by the autochthonous bacterial and yeast isolates. Microorganisms.

[63] Mason CJ, Pfammatter JA, Holeski LM, Raffa KF (2015). Foliar bacterial communities of trembling aspen in a common garden. Can J Microbiol.

[64] Rastogi G, Sbodio A, Tech JJ, Suslow TV, Coaker GL, Leveau JHJ (2012). Leaf microbiota in an agroecosystem: spatiotemporal variation in bacterial community composition on field-grown lettuce. ISME J.

[65] Wu H, Zhang Z, Wang J, Qin X, Chen J, Wu L (2022). Bio-fertilizer amendment alleviates the replanting disease under consecutive monoculture regimes by reshaping leaf and root microbiome. Microb Ecol.

[66] Yashiro E, McManus PS (2012). Effect of streptomycin treatment on bacterial community structure in the apple phyllosphere. PLoS ONE.

[67] Bian G-K, Feng Z-Z, Qin S, Xing K, Wang Z, Cao C-L (2012). *Kineococcus endophytica* sp. nov., a novel endophytic actinomycete isolated from a coastal halophyte in Jiangsu China. Antonie Leeuwenhoek.

[68] Duangmal K, Thamchaipenet A, Ara I, Matsumoto A, Takahashi Y (2008). *Kineococcus gynurae* sp. nov., isolated from a Thai medicinal plant. Int J Syst Evol Microbiol.

[69] Lee SD (2009). Kineococcus rhizosphaerae sp. nov., isolated from rhizosphere soil. Int J Syst Evol Microbiol.

[70] Qin S, Bian G-K, Zhang Y-J, Xing K, Cao C-L, Liu C-H (2013). *Modestobacter roseus* sp. nov., an endophytic actinomycete isolated from the coastal halophyte* Salicornia europaea* Linn., and emended description of the genus* Modestobacter*. Int J Syst Evol Microbiol.

[71] Busarakam K, Bull AT, Trujillo ME, Riesco R, Sangal V, van Wezel GP (2016). *Modestobacter caceresii* sp. nov., novel actinobacteria with an insight into their adaptive mechanisms for survival in extreme hyper-arid Atacama Desert soils. Syst Appl Microbiol.

[72] Molina-Menor E, Gimeno-Valero H, Pascual J, Peretó J, Porcar M (2020). Kineococcus vitellinus sp. nov., Kineococcus indalonis sp nov and Kineococcus siccus sp. nov., isolated nearby the Tabernas Desert (Almería, Spain). Microorganisms.

[73] Xu F-J, Li Q-Y, Li G-D, Chen X, Jiang Y, Jiang C-L (2017). *Kineococcus terrestris* sp. nov. and* Kineococcus aureolus* sp. nov., isolated from saline sediment. Int J Syst Evol Microbiol.

[74] Makhalanyane TP, Valverde A, Gunnigle E, Frossard A, Ramond J-B, Cowan DA (2015). Microbial ecology of hot desert edaphic systems. FEMS Microbiol Rev.

[75] Mohammadipanah F, Wink J (2016). *Actinobacteria* from arid and desert habitats: diversity and biological activity. Front Microbiol.

[76] Hansen TE, Enders LS (2022). Host plant species influences the composition of milkweed and monarch microbiomes. Front Microbiol.

[77] Li S-J, Ahmed MZ, Lv N, Shi P-Q, Wang X-M, Huang J-L (2017). Plant-mediated horizontal transmission of *Wolbachia* between whiteflies. ISME J.

[78] Sabree ZL, Huang CY, Okusu A, Moran NA, Normark BB (2013). The nutrient supplying capabilities of *Uzinura*, an endosymbiont of armoured scale insects. Environ Microbiol.

[79] Besaury L, Rémond C (2022). Culturable and metagenomic approaches of wheat bran and wheat straw phyllosphere’s highlight new lignocellulolytic microorganisms. Lett Appl Microbiol.

[80] Safni I, Subandiyah S, Fegan M (2018). Ecology, epidemiology and disease management of *Ralstonia syzygii* in Indonesia. Front Microbiol.

[81] Jackson CR, Denney WC (2011). Annual and seasonal variation in the phyllosphere bacterial community associated with leaves of the southern magnolia (*Magnolia grandiflora*). Microb Ecol.

[82] Kim M, Singh D, Lai-Hoe A, Go R, Abdul Rahim R, Ainuddin A (2012). Distinctive phyllosphere bacterial communities in tropical trees. Microb Ecol.

[83] Lambais MR, Lucheta AR, Crowley DE (2014). Bacterial community assemblages associated with the phyllosphere, dermosphere, and rhizosphere of tree species of the Atlantic Forest are host taxon dependent. Microb Ecol.

[84] Hiraishi A, Imhoff JF, Whitman WB, Rainey F, Kämpfer P, Trujillo M, Chun J, DeVos P, Hedlund B, Dedysh S (2015). Acidiphilium. Bergey’s Manual of Systematics of Archaea and Bacteria.

[85] Knief C, Ramette A, Frances L, Alonso-Blanco C, Vorholt JA (2010). Site and plant species are important determinants of the *Methylobacterium* community composition in the plant phyllosphere. ISME J.

[86] Mina D, Pereira JA, Lino-Neto T, Baptista P (2020). Epiphytic and endophytic bacteria on olive tree phyllosphere: exploring tissue and cultivar effect. Microb Ecol.

[87] Schlechter RO, Miebach M, Remus-Emsermann MNP (2019). Driving factors of epiphytic bacterial communities: a review. J Adv Res.

[88] Kearl J, McNary C, Lowman JS, Mei C, Aanderud ZT, Smith ST (2019). Salt-tolerant halophyte rhizosphere bacteria stimulate growth of alfalfa in salty soil. Front Microbiol.

[89] Manni A, Filali-Maltouf A (2022). Diversity and bioprospecting for industrial hydrolytic enzymes of microbial communities isolated from deserted areas of South-East Morocco. AIMS Microbiol.

[90] Osman JR, Wang Y, Jaubert C, Nguyen T-N, Fernandes GR, DuBow MS (2021). The bacterial communities of surface soils from desert sites in the eastern Utah (USA) portion of the Colorado Plateau. Microbiol Res.

[91] Palmisano MM, Nakamura LK, Duncan KE, Istock CA, Cohan FM (2001). Bacillus sonorensis sp nov, a close relative of Bacillus licheniformis, isolated from soil in the Sonoran Desert Arizona. Int J Syst Evol Microbiol.

[92] Xie F, Pathom-aree W (2021). *Actinobacteria* from desert: diversity and biotechnological applications. Front Microbiol.

[93] Khan N, Bano A, Babar MDA (2017). The root growth of wheat plants, the water conservation and fertility status of sandy soils influenced by plant growth promoting rhizobacteria. Symbiosis.

[94] Mukhtar S, Mehnaz S, Malik KA (2021). Comparative study of the rhizosphere and root endosphere microbiomes of Cholistan Desert plants. Front Microbiol.

[95] Radwan SS, Al-Awadhi H, Sorkhoh NA, El-Nemr IM (1998). Rhizospheric hydrocarbon-utilizing microorganisms as potential contributors to phytoremediation for the oil Kuwaiti Desert. Microbiol Res.

[96] Safdarian M, Askari H, Nematzadeh G, Sofo A (2020). Halophile plant growth-promoting rhizobacteria induce salt tolerance traits in wheat seedlings (Triticum aestivum L.). Pedosphere.

[97] Shahid M, Akram MS, Khan MA, Zubair M, Shah SM, Ismail M (2018). A phytobeneficial strain Planomicrobium sp. MSSA-10 triggered oxidative stress responsive mechanisms and regulated the growth of pea plants under induced saline environment. J Appl Microbiol.

[98] Beary TP, Boopathy R, Templet P (2002). Accelerated decomposition of sugarcane crop residue using a fungal–bacterial consortium. Int Biodet Biodegrad.

[99] Calleja-Cervantes ME, Menéndez S, Fernández-González AJ, Irigoyen I, Cibriáin-Sabalza JF, Toro N (2015). Changes in soil nutrient content and bacterial community after 12 years of organic amendment application to a vineyard. Eur J Soil Sci.

[100] Fan F, Yin C, Tang Y, Li Z, Song A, Wakelin SA (2014). Probing potential microbial coupling of carbon and nitrogen cycling during decomposition of maize residue by ^13^C-DNA-SIP. Soil Biol Biochem.

[101] Kramer S, Dibbern D, Moll J, Huenninghaus M, Koller R, Krueger D (2016). Resource partitioning between bacteria, fungi, and protists in the detritusphere of an agricultural soil. Front Microbiol.

[102] Maarastawi SA, Frindte K, Geer R, Kröber E, Knief C (2018). Temporal dynamics and compartment specific rice straw degradation in bulk soil and the rhizosphere of maize. Soil Biol Biochem.

[103] Latorre C, González AL, Quade J, Fariña JM, Pinto R, Marquet PA (2011). Establishment and formation of fog-dependent *Tillandsia landbeckii* dunes in the Atacama Desert: evidence from radiocarbon and stable isotopes. J Geophys Res Biogeosci.

[104] Fuentes B, Choque A, Gomez F, Alarcon J, Castro-Nallar E, Arenas F (2022). Influence of physical-chemical soil parameters on microbiota composition and diversity in a deep hyperarid core of the Atacama Desert. Front Microbiol.

[105] Scola V, Ramond JB, Frossard A, Zablocki O, Adriaenssens EM, Johnson RM (2018). Namib Desert soil microbial community diversity, assembly, and function along a natural xeric gradient. Microb Ecol.

[106] Bodenhausen N, Bortfeld-Miller M, Ackermann M, Vorholt JA (2014). A synthetic community approach reveals plant genotypes affecting the phyllosphere microbiota. PLoS Genet.

[107] Morella NM, Weng FC-H, Joubert PM, Metcalf CJE, Lindow S, Koskella B (2020). Successive passaging of a plant-associated microbiome reveals robust habitat and host genotype-dependent selection. Proc Natl Acad Sci USA.

[108] Wagner MR, Lundberg DS, del Rio TG, Tringe SG, Dangl JL, Mitchell-Olds T (2016). Host genotype and age shape the leaf and root microbiomes of a wild perennial plant. Nat Commun.

[109] Wallace JG, Kremling KA, Kovar LL, Buckler ES (2019). Quantitative genetics of the maize leaf microbiome. Phytobiomes J.

[110] Durán P, Ellis TJ, Thiergart T, Ågren J, Hacquard S (2022). Climate drives rhizosphere microbiome variation and divergent selection between geographically distant *Arabidopsis* populations. New Phytol.

[111] Laforest-Lapointe I, Messier C, Kembel SW (2016). Host species identity, site and time drive temperate tree phyllosphere bacterial community structure. Microbiome.

[112] García J-L, Lobos-Roco F, Schween JH, del Río C, Osses P, Vives R (2021). Climate and coastal low-cloud dynamic in the hyperarid Atacama fog Desert and the geographic distribution of *Tillandsia landbeckii* (*Bromeliaceae*) dune ecosystems. Plant Syst Evol.

[113] Koch MA, Stock C, Kleinpeter D, del Río C, Osses P, Merklinger FF (2020). Vegetation growth and landscape genetics of *Tillandsia* lomas at their dry limits in the Atacama Desert show fine-scale response to environmental parameters. Ecol Evol.

