# Supplementary material for: Tillandsia landbeckii phyllosphere and laimosphere as refugia for bacterial life in a hyperarid desert environment
Source: Microbiome. 2023 Nov 8;11:246. doi: 10.1186/s40168-023-01684-x (PMC10631034; doi:10.1186/s40168-023-01684-x)
Supplement: Supplementary file 2 — Additional file 1: Supplementray figures: Fig. S1. Principal component plot of surface soil samples showing the variation in bacterial community composition between samples from different study sites. DNA extraction negative control (EC) and PCR negative control (EC) are included. They both remained slightly positive after applying a decontamination algorithm and cluster with surface soil samples from some, though not all sampling sites. Fig. S2. Principal component plots showing the variation in bacterial community composition in phyllosphere and laimosphere samples from living and dead plant material, collected at Salar Grande and Cerro Pajonal. The tables below the figures summarize PERMANOVA results. Fig. S3. Heat trees showing the bacterial community structure up to genus level in the complete datasets of the phyllosphere and laimosphere. The color and size of nodes and edges are correlated with the sum of the mean abundance and number of ASVs, respectively. The most abundant genera (> 10% abundance in individual samples) are highlighted. The abundance value of 0.001 was set as a threshold to remove low-abundance taxa and reduce the complexity of the plots. Fig. S4. Bacterial diversity in the phyllosphere and laimosphere of T. landbeckii. Shown are Shannon’s index (upper row), evenness (middle row) and Faith’s PD (lower row). Significant differences in dependence on study site were evaluated based on Kruskal-Wallis tests. Please note the different y-axis scales for phyllosphere and laimosphere. Fig. S5. Bacterial diversity in the phyllosphere in dependence on the season for the samples from four different study sites. Shown are Shannon’s index, evenness, and Faith’s PD. Significant differences in dependence on the season were evaluated based on Kruskal-Wallis tests and are reported above the respective panel. Pairwise comparisons within each study site were done using Wilcoxon signed rank exact test for the paired samples and the Wilcoxon sum exact test for non [file 40168_2023_1684_MOESM1_ESM.pdf]

Supplementary material

Tillandsia landbeckii phyllosphere and laimosphere as refugia for bacterial life in a hyperarid desert environment

Anna Hakobyan, Stefanie Velte, Wiebke Sickel, Dietmar Quandt, Alexandra Stoll, Claudia Knief

Supplementary figures

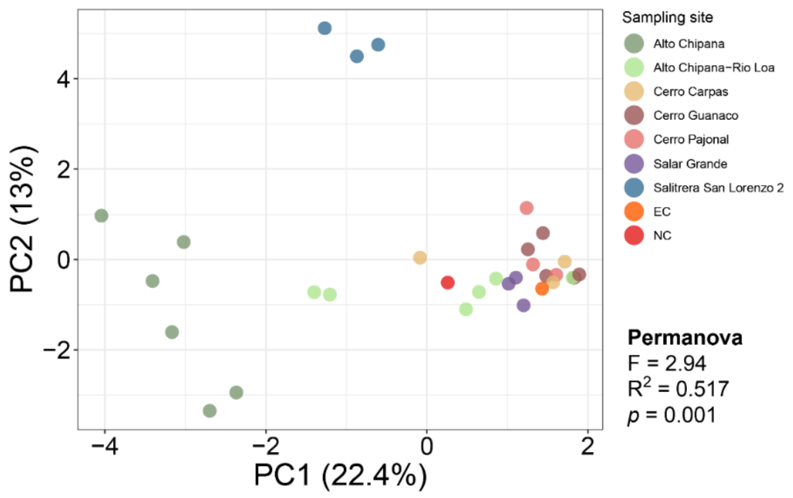

Figure S1. Principal component plot of surface soil samples showing the variation in bacterial community composition between samples from different study sites. DNA extraction negative control (EC) and PCR negative control (EC) are included. They both remained slightly positive after applying a decontamination algorithm and cluster with surface soil samples from some, though not all sampling sites.

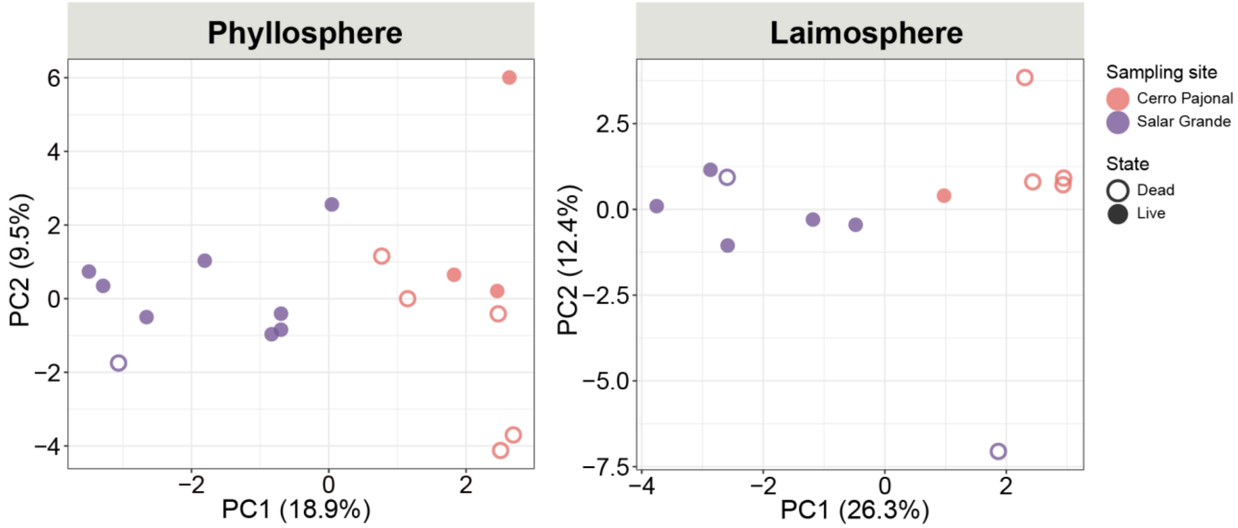

PERMANOVA (Phyllosphere)

Factor	F	R^2	*p*
Site	7.765	0.324	0.001
Live/dead (LD)	1.237	0.052	0.281
Site/LD	1.990	0.083	0.066

PERMANOVA (Laimosphere)

Factor	F	R^2	*p*
Site	5.518	0.320	0.004
Live/dead (LD)	2.530	0.147	0.035
Site/LD	1.201	0.070	0.282

Figure S2. Principal component plots showing the variation in bacterial community composition in phyllosphere and laimosphere samples from living and dead plant material, collected at Salar Grande and Cerro Pajonal. The tables below the figures summarize Permanova results.

Phyllosphere

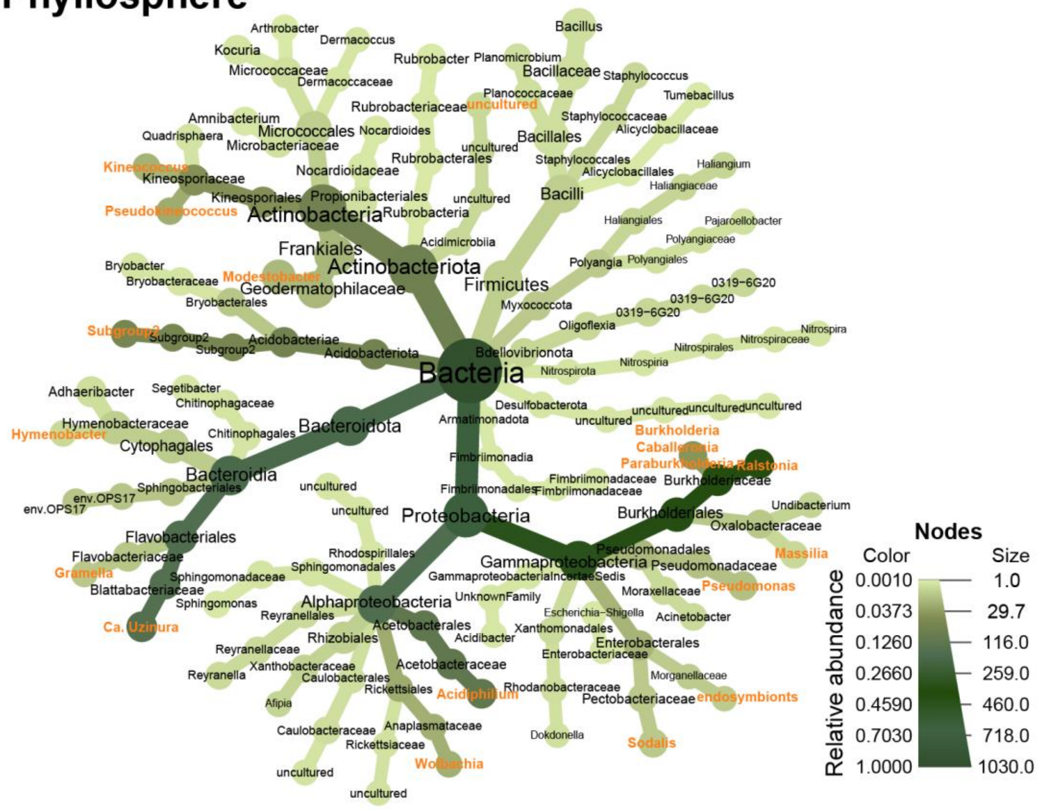

Laimosphere

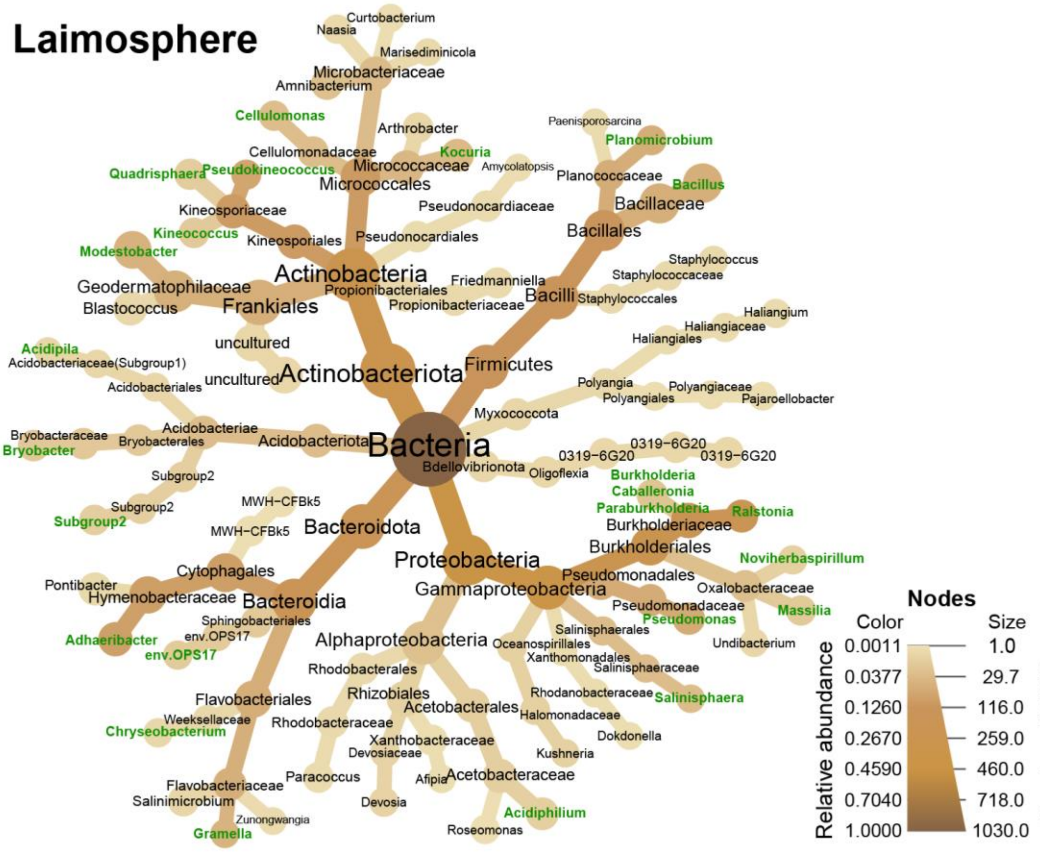

Figure S3. Heat trees showing the bacterial community structure up to genus level in the complete datasets of the phyllosphere and laimosphere. The color and size of nodes and edges are correlated with the sum of the mean abundance and number of ASVs, respectively. The most abundant genera (>10% abundance in individual samples) are highlighted. The abundance value of 0.001 was set as a threshold to remove low-abundance taxa and reduce the complexity of the plots.

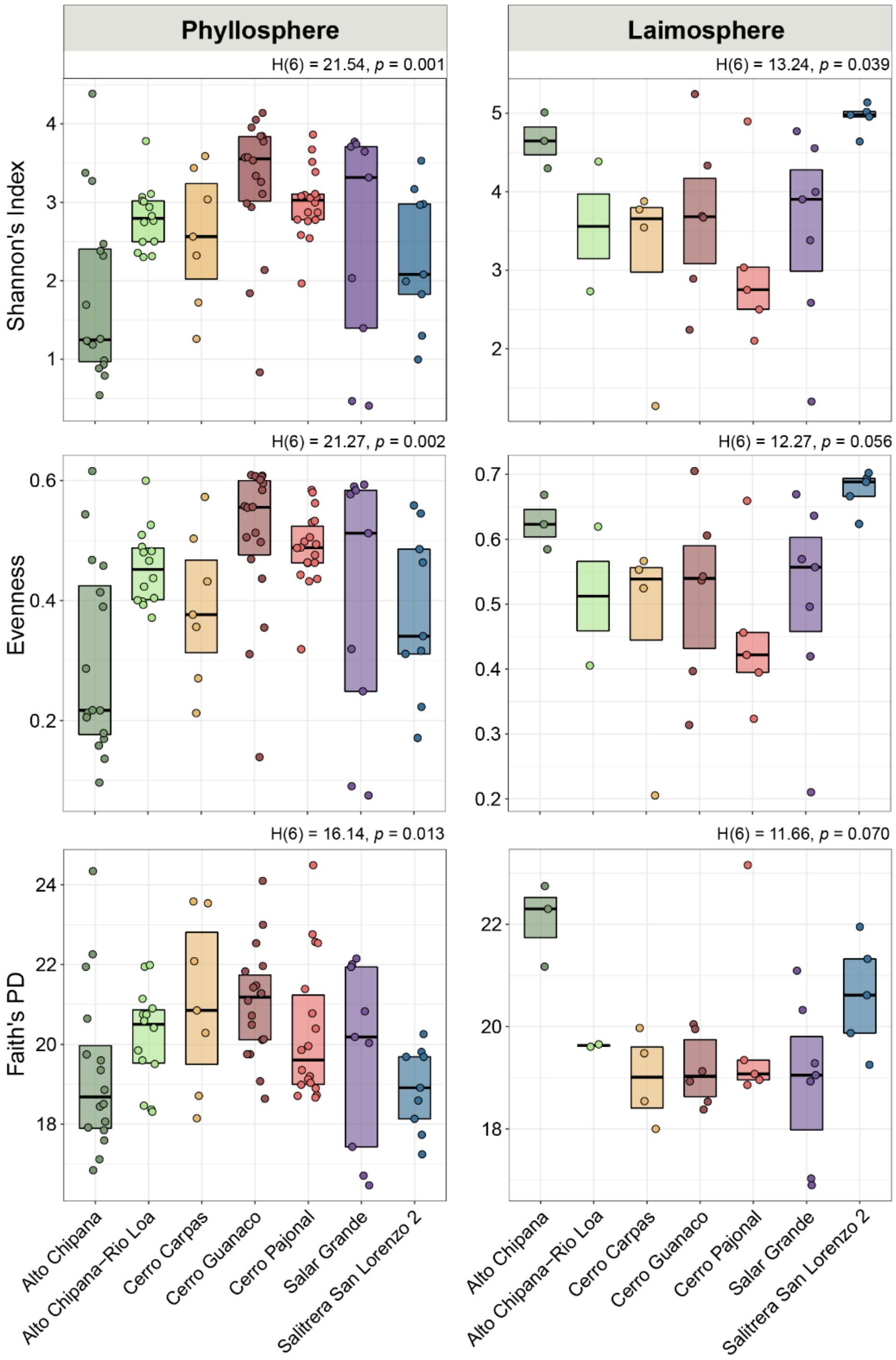

Figure S4. Bacterial diversity in the phyllosphere and laimosphere of *T. landbeckii*. Shown are Shannon's index (upper row), evenness (middle row) and Faith's PD (lower row). Significant differences in dependence on study site were evaluated based on Kruskal-Wallis tests. Please note the different y-axis scales for phyllosphere and laimosphere.

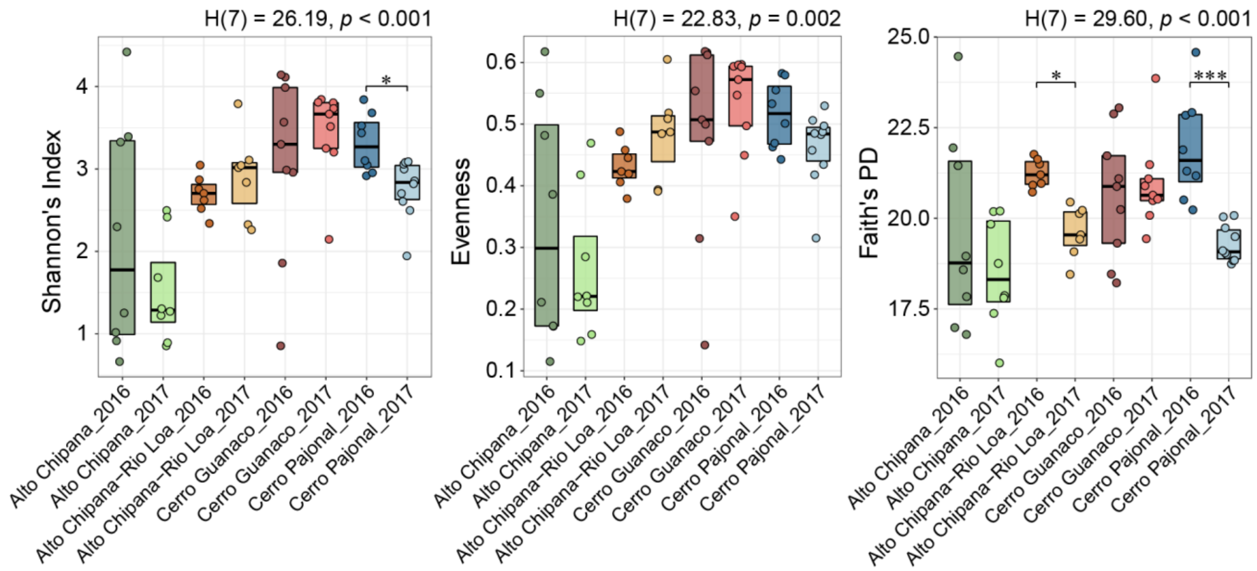

Figure S5. Bacterial diversity in the phyllosphere in dependence on the season for the samples from four different study sites. Shown are Shannon's index, evenness, and Faith's PD. Significant differences in dependence on the season were evaluated based on Kruskal-Wallis tests and are reported above the respective panel. Pairwise comparisons within each study site were done using Wilcoxon signed rank exact test for the paired samples and the Wilcoxon sum exact test for non-paired samples (e.g. Cerro Pajonal 2016 vs. 2017). Significance values: *$p < 0.05$, ***$p < 0.001$.
